# Classification of Pneumonia, Tuberculosis and Covid-19 from Chest X-Ray Images Using Convolution Neural Network Model

**DOI:** 10.5539/ijsp.v13n4p42

**Published:** 2024-11-30

**Authors:** J Kiche, Ivivi Mwaniki, Idah Orowe, Patrick Weke

**Affiliations:** 1 Mathematics Department, University of Nairobi,Kenya.; 2 Mathematics Department, University of Nairobi, Kenya.; 3 Mathematics Department, University of Nairobi,Kenya.; 4 Mathematics Department, University of Nairobi,Kenya.

**Keywords:** CNN-Convolutional Neural Networks, AUC-ROC-Area Under the Receiver Operating Characteristic Curve, PMI-Pointwise Mutual Information

## Abstract

Accurate and timely diagnosis of respiratory ailments like pneumonia, tuberculosis (TB), and COVID-19 is pivotal for effective patient care and public health interventions. Deep learning algorithms have emerged as potent tools in medical image classification, offering promise for automated diagnosis and screening. This study presents a deep learning-based approach for categorizing chest X-ray images into three classes: pneumonia, tuberculosis, and COVID-19. Utilizing convolutional neural networks (CNNs) as the primary architecture, owing to their ability to automatically extract relevant features from raw image data. The proposed model is trained on a sizable dataset of chest X-ray images annotated with ground truth labels for pneumonia, TB, and COVID-19. Extensive experiments are conducted to evaluate the model’s performance in terms of classification accuracy, sensitivity, specificity, and area under the receiver operating characteristic curve (AUC-ROC). Additionally, we compare the performance of our deep learning model with traditional machine learning techniques, including support vector machines, decision trees, XGBoost, and evaluate its performance on an independent test set. Our findings demonstrate that the proposed deep learning model achieves high accuracy in classifying chest X-ray images of pneumonia, TB, and COVID-19, outperforming traditional methods and showing potential for clinical deployment as a screening tool, especially in resource-limited settings.

## 1. Introduction

Respiratory conditions like pneumonia, tuberculosis (TB), and COVID-19 pose significant global health challenges due to their impact on morbidity and mortality rates. Prompt and accurate diagnosis is essential for effectively managing these diseases. Although chest X-ray imaging is commonly used for detecting lung abnormalities associated with respiratory ailments, interpretation requires expertise and can lead to delays in diagnosis, especially in resource-limited settings. In recent years, deep learning algorithms, particularly convolutional neural networks (CNNs), have demonstrated superior performance in various medical image analysis tasks, including classification, segmentation, and detection. These algorithms excel at extracting relevant features from raw image data, making them well-suited for tasks like disease classification in medical images. To address the need for precise and efficient diagnosis of respiratory illnesses, researchers have increasingly explored deep learning models for analyzing chest X-ray images. By leveraging large datasets of annotated images, these models can differentiate between different respiratory conditions, including pneumonia, TB, and COVID-19. This study contributes to the field by proposing a deep learning-based approach tailored for classifying chest X-ray images into three categories: pneumonia, TB, and COVID-19. By leveraging CNNs and annotated datasets, the goal is to develop a reliable and accurate classification model to aid healthcare professionals in swiftly and accurately identifying respiratory diseases from chest X-ray images. The ultimate objective is to improve diagnostic efficiency, enable timely interventions, and enhance patient outcomes, particularly in regions with limited access to specialized healthcare resources.

## 2. Global and Regional Insights into COVID-19, Tuberculosis, and Pneumonia

As of mid-2024, the global impact of COVID-19 has substantially declined from its peak, with marked reductions in both case numbers and mortality rates. Cumulatively, over 687 million infections and approximately 6.9 million deaths have been reported globally, with vaccination programs playing a critical role in curbing the pandemics severity and spread. Billions of vaccine doses have been administered, though disparities in coverage remain. In Africa, the pandemic has resulted in more than 12 million confirmed cases and approximately 255,000 deaths. However, vaccine accessibility, logistical hurdles, and hesitancy continue to challenge the regions public health efforts. Kenya, with about 340,000 reported cases and 5,700 fatalities, has made commendable progress in its vaccination campaign, though efforts to address emerging variants and expand coverage are ongoing.

Tuberculosis (TB) remains a critical infectious disease globally, affecting millions annually. In 2022 alone, 10.6 million new TB cases and 1.6 million deaths were documented, with the disease disproportionately impacting low- and middle-income nations. Challenges such as drug-resistant TB strains and gaps in healthcare infrastructure exacerbate the situation, particularly in high-burden countries. Africa carries a significant share of the TB burden, accounting for a quarter of the global caseload. Contributing factors include high rates of HIV co-infection and limited healthcare resources. Kenya, identified among the top 30 countries with the highest TB burdens, reported approximately 140,000 cases in 2022. Enhanced diagnostics, expanded treatment access, and initiatives targeting drug-resistant TB have yielded positive results, though TB-HIV co-infection remains a pressing issue.

Pneumonia, a major cause of death among children under five, accounted for roughly 740,000 fatalities in this age group in 2022. Its prevalence is linked to factors such as malnutrition, inadequate vaccination coverage, and substandard living conditions. Sub-Saharan Africa continues to bear a disproportionate share of the global burden, despite ongoing interventions. In Kenya, pneumonia significantly impacts child health, with vaccination initiatives and improved healthcare access contributing to reductions in mortality. However, the disease persists as a leading cause of morbidity among young children, requiring sustained public health interventions. These respiratory illnesses, while distinct, share common challenges, including healthcare inequities and the need for robust vaccination and treatment programs. Globally and within regions like Africa and Kenya, continued vigilance and innovation are essential to mitigate their impacts and improve health outcomes for affected populations.

In Africa, pneumonia accounts for an annual incidence of 38 million cases and 900,000 deaths, highlighting its significant impact on public health. Tuberculosis in Africa has an annual incidence of 2.9 million cases and causes 450,000 deaths each year, underscoring its persistent public health challenge. In 2024, COVID-19 in Africa resulted in 9 million cases and 180,000 deaths, reflecting its ongoing impact on the region’s health systems.

In Kenya, pneumonia accounts for 1.4 million cases annually and causes 45,000 deaths each year, representing a significant public health concern. In Kenya, tuberculosis affects 135,000 individuals annually and results in 23,000 deaths each year, highlighting its substantial burden on public health. In 2024, Kenya reported 450,000 COVID-19 cases and 9,000 deaths, emphasizing the continued impact of the pandemic on the country’s health systems.

The statistics from 2024 highlight the ongoing challenges posed by pneumonia, tuberculosis, and COVID-19 at global, regional (Africa), and national (Kenya) levels. While global efforts continue to combat these diseases, the burden remains particularly high in Africa and Kenya. Addressing these health issues requires targeted interventions, increased healthcare funding, and robust public health strategies to reduce incidence and mortality rates.

## 3. Types of Learning as used in Machine Learning

### 3.1 Supervised Learning

CNNs are predominantly utilized in supervised learning, where the model is trained on labeled data, meaning each input is paired with a corresponding output label. CNNs are particularly effective in image-related tasks, including image classification, object detection, and segmentation. In image classification, the goal is to assign a label to an entire image from a predefined set of categories. Object detection involves identifying and locating specific objects within an image, while segmentation focuses on classifying each pixel of an image into distinct categories. Through exposure to labeled training data, CNNs learn to identify patterns and features that correspond to the provided labels, enabling them to perform these tasks with high accuracy.

### 3.2 Unsupervised Learning

CNNs are primarily associated with supervised learning, they can also be applied to unsupervised learning tasks, albeit less commonly. In unsupervised learning, the model is trained on data without explicit labels, focusing instead on uncovering patterns or structures within the data. CNNs are employed in tasks such as autoencoders and Generative Adversarial Networks (GANs). CNN-based autoencoders are used for image compression and reconstruction by learning an efficient representation of the input data. CNNs are also used in GANs to generate new data that resembles the training set distribution.”

### 3.3 Semi-Supervised and Self-Supervised Learning

Semi-supervised and self-supervised learning are hybrid approaches where CNNs can be effectively utilized. In semi-supervised learning, a small amount of labeled data is combined with a large amount of unlabeled data during training, allowing CNNs to leverage both to enhance performance. Self-supervised learning, on the other hand, generates supervisory signals from the data itself, such as predicting the rotation of an image or filling in missing parts, providing supervision without the need for labeled data. While CNNs are most commonly used in supervised learning tasks, they can also be adapted for unsupervised learning and other hybrid learning paradigms. However, their primary strength and most widespread application remain in scenarios where labeled data is available.

## 4. Methodology

1. Dataset Acquisition and Preprocessing Dataset Selection: A comprehensive and well-annotated dataset of chest X-ray images encompassing cases of pneumonia, tuberculosis (TB), and COVID-19 were sourced from reputable repositories or collaborations with healthcare institutions.
  Data Augmentation: To mitigate data scarcity and enhance model generalization, various data augmentation techniques were applied. This include random rotations, horizontal and vertical flipping, as well as slight adjustments in brightness and contrast.
  Data Splitting: The dataset hadl undergone the stratified splitting into three subsets: a training set (88.66%), a validation set (0.53%), and a testing set (10.81% ).
2. Image Preprocessing Image Resizing: All images were uniformly resized to dimensions of (224x224 pixels) to ensure consistency and compatibility with the chosen CNN architecture.
  Normalization: Pixel intensity values were scaled to a range between 0 and 1 to facilitate model convergence and enhance training efficiency.
3. CNN Architecture Model Selection: The DenseNet121 architecture, a pre-trained CNN model known for its effectiveness in image classification tasks, were chosen as the base model.
  Transfer Learning: The pre-trained DenseNet121 model were initialized with weights from the ImageNet dataset. The final layers were then modified to suit the three-class classification task.
  Fine-tuning: The model had undergone fine-tuning using the training set, allowing it to adapt to the specific features of pneumonia, TB, and COVID-19 in chest X-ray images.
  Layer Modification: The final classification layer were adjusted to output probabilities for the three target classes using a softmax activation function.
4. Model Training Hyperparameter Tuning: Hyperparameters such as learning rate, batch size, and optimizer (Adam) settings were optimized using a grid search technique combined with cross-validation on the training set.
  Training Process: The model were trained on the training set with early stopping based on validation loss to prevent overfitting. Training iterations were set to 30 epochs.
  Class Imbalance Handling: To address the class imbalance issue, class weights were applied during training to give more weight to minority classes (TB and COVID-19).
5. Model Evaluation Performance Metrics: The model’s performance was evaluated on both the validation and testing sets using standard metrics such as accuracy, precision, recall, F1-score, and confusion matrices.
  Comparison: The model’s performance were compared against baseline models and state-of-the-art methods reported in the literature for similar tasks.
6. Interpretability and Visualization Interpretability Techniques: Techniques such as Grad-CAM (Gradient-weighted Class Activation Mapping) were employed to visualize and interpret the CNN model’s decision-making process.
  Feature Importance: Feature maps were generated to identify key regions in chest X-ray images that significantly influenced the classification decisions.

### 4.1 CNN Model for Image Classification

The CNN model for image classification begins with an input layer that accepts raw pixel data of the image, where, for a color image of size 32x32 pixels, the input dimension is 32x32x3 (height, width, and color channels). The first convolutional layer applies 32 filters of size 3x3 with ReLU activation, resulting in 32 feature maps of size 30x30. This is followed by a max-pooling layer with a pool size of 2x2, reducing the output to 32 feature maps of size 15x15. The second convolutional layer uses 64 filters of size 3x3 with ReLU activation, producing 64 feature maps of size 13x13, which are then passed through another max-pooling layer with a 2x2 pool size, resulting in 64 feature maps of size 6x6. The third convolutional layer applies 128 filters of size 3x3 with ReLU activation, generating 128 feature maps of size 4x4, which are then reduced by a max-pooling layer of 2x2, resulting in 128 feature maps of size 2x2. The flatten layer converts these 2D feature maps into a 1D feature vector of 512 neurons (128 x 2 x 2). This is followed by a fully connected layer with 512 neurons and ReLU activation. A dropout layer with a dropout rate of 0.5 is applied to prevent overfitting. Finally, a fully connected layer with 4 neurons (with 4 classes for classification) and softmax activation produces a probability distribution over the class labels.

### 4.2 Filtering (Convolution)

In CNNs, filtering refers to the process of applying convolutional filters (or kernels) to an input image or feature map. This process involves sliding the filter across the input and computing the dot product between the filter weights and the input values covered by the filter. The result is a new feature map that highlights specific patterns, such as edges or textures, depending on the filter’s design.

Given an input image I of dimensions H×W and a filter K of dimensions f×f, the convolution operation can be defined as:

(1) $$\left(I*K\right)\left(i,j\right)=\sum_{m=1}^{f}\sum_{n=1}^{f}I\left(i+m-1,j+n-1\right)\cdot K\left(m,n\right)$$

where $\left(i,j\right)$ denotes the position in the output feature map.

### 4.3 Padding in Convolutional Neural Networks

Padding is the practice of adding extra pixels around the edges of an input image or feature map to influence the spatial dimensions of the output feature map after a convolution operation. This technique is essential for preserving the spatial dimensions of the input, reducing information loss, and ensuring that edge pixels contribute equally to the computation. It also provides flexibility in controlling the size of the output, which is particularly important in deeper networks.

There are different approaches to padding, each serving specific purposes. One common method is zero padding, where pixels with a value of zero are added around the input. This helps maintain the spatial dimensions of the original image while incorporating information from edge pixels. Another approach is same padding, which ensures the dimensions of the output feature map match those of the input feature map by adjusting the padding appropriately. Conversely, valid padding involves no additional pixels, resulting in a smaller output feature map since only valid regions of the input are used for convolution.

Mathematically, for an input image I with height H and width W, and a padding size p, the padded image $I_{\text{padded}}$ can be expressed as:

(2) $$I_{\text{padded}}\left(i,j\right)=\left\{\begin{array}{ll} 0 & \text{if}\;i\leq p\;\text{or}\;j\leq p\;\text{or}\;i>H+p\;\text{or}\;j>W+p\\ I\left(i-p,j-p\right) & \text{otherwise} \end{array}\right.$$

where H and W are the height and width of the original image.

This formulation ensures that the input dimensions can be adjusted as needed, while edge pixels are included in the convolution operation, preserving important spatial information.

Given an input image I of dimensions H×W and a filter K of dimensions f×f, padding p pixels around the image changes the dimensions of the input to $\left(H+2p\right)\times \left(W+2p\right)$.

The output feature map dimensions are given by:

$$H_{\text{out}}=\frac{H+2p-f}{s}+1$$

$$W_{\text{out}}=\frac{W+2p-f}{s}+1$$

where s is the stride of the convolution.

### 4.4 Pooling in Convolutional Neural Networks

Pooling is a down-sampling operation that reduces the spatial dimensions (height and width) of the input feature maps, helping to reduce computational complexity, control overfitting, and achieve spatial invariance. There are different types of pooling: Max pooling selects the maximum value from each patch of the feature map, Average pooling calculates the average value from each patch, and Global pooling reduces the entire feature map to a single value by taking the maximum or average of all values in the feature map. Pooling is used for dimensionality reduction, as it decreases the number of parameters and computations in the network, promotes spatial invariance, making the network more resistant to small translations of the input image, and helps control overfitting by summarizing features. Pooling operations reduce the dimensions of the feature maps. For max pooling with a 2 × 2 window and stride 2, the operation can be written as:

(3) $$P\left(i,j\right)=\max\left(\begin{array}{cc} I\left(2i-1,2j-1\right) & I\left(2i-1,2j\right)\\ I\left(2i,2j-1\right) & I\left(2i,2j\right) \end{array}\right)$$

where P is the pooled feature map.

Pooling is a down-sampling operation used in convolutional neural networks (CNNs) to reduce the spatial dimensions of the input feature maps. It helps in reducing computational complexity, controlling overfitting, and achieving spatial invariance. Pooling layers reduce the spatial dimensions of the input. Max pooling selects the maximum value from each patch of the feature map, while average pooling calculates the average value from each patch. Global pooling, on the other hand, reduces the entire feature map to a single value by taking either the maximum or the average of all values in the feature map. Max pooling operation on an input I with a pooling window of size p×p and stride s is defined as:

(4) $$O\left(i,j\right)=\underset{0\leq m,n<p}{\text{max}}I\left(s\cdot i+m,s\cdot j+n\right)$$

Average pooling operation on an input I with a pooling window of size p×p and stride s is defined as:

(5) $$O\left(i,j\right)=\frac{1}{p^{2}}\sum_{m=0}^{p-1}\sum_{n=0}^{p-1}I\left(s\cdot i+m,s\cdot j+n\right)$$

Consider an input feature map of dimensions H×W with a pooling window of size p×p and a stride s. The dimensions of the output feature map after applying the pooling operation are given by:

$$H_{\text{out}}=\left\lfloor \frac{H-p}{s}\right\rfloor +1$$

$$W_{\text{out}}=\left\lfloor \frac{W-p}{s}\right\rfloor +1$$

### 4.5 Convolutional Layer

The convolutional layer is the core building block of a CNN. The layer’s parameters consist of a set of learnable filters (or kernels), which have a small receptive field but extend through the full depth of the input volume. Consider an input image I of dimensions H×W×D (height, width, depth), and a filter K of dimensions f×f×D (filter size f, same depth as the input). The output O of a convolution operation with stride s and padding p is given by:

(6) $$O\left(i,j\right)=\sum_{m=0}^{f-1}\sum_{n=0}^{f-1}\sum_{d=0}^{D-1}I\left(i+m,j+n,d\right)\cdot K\left(m,n,d\right)$$

where i and j are the spatial coordinates of the output.

The dimensions of the output feature map are given by:

$$H_{\text{out}}=\left\lfloor \frac{H-f+2p}{s}\right\rfloor +1$$

$$W_{\text{out}}=\left\lfloor \frac{W-f+2p}{s}\right\rfloor +1$$

After several convolutional and pooling layers, the high-level reasoning in the neural network is done via fully connected (FC) layers. These layers take the input from the final pooling or convolutional layer and output a class score.

The output y of a fully connected layer with input x and weights W is given by:

y=Wx+b

where W is the weight matrix and b is the bias vector.

### 4.6 Loss Function

For a classification problem, the cross-entropy loss is commonly used:

$$L=\;-\sum_{i}y_{i}\text{log}\left({\hat{y}}_{i}\right)$$

where $y_{i}$ is the true label and ${\hat{y}}_{i}$ is the predicted probability for class i. During training, CNNs use backpropagation to compute gradients of the loss function with respect to the weights. This involves the chain rule of calculus to propagate the error derivatives back through the network.

Convolutional Neural Networks are powerful models for image classification, detection, and many other tasks. Their ability to automatically learn spatial hierarchies of features makes them well-suited for tasks involving image and speech processing.

### 4.7 Activation Functions in Deep Learning

Activation functions play a crucial role in introducing non-linearity to neural networks, allowing them to learn complex patterns and relationships in data. Here are some common activation functions used in deep learning: The sigmoid activation function, also known as the logistic function, is defined as:

(7) $$\sigma \left(x\right)=\frac{1}{1+e^{-x}}$$

The sigmoid function squashes the input x to the range (0, 1), making it suitable for binary classification tasks and as an output layer activation for binary classification problems.

The hyperbolic tangent activation function, tanh, is defined as:

(8) $$\text{tanh}\left(x\right)=\frac{e^{x}-e^{-x}}{e^{x}+e^{-x}}$$

The tanh function squashes the input x to the range (−1, 1), providing stronger gradients than the sigmoid function. It is commonly used in hidden layers of neural networks.

The Rectified Linear Unit (ReLU) is a widely used activation function defined as:

(9) $$\text{ReLU}\left(x\right)=\text{max}\left(0,x\right)$$

ReLU is computationally efficient and helps mitigate the vanishing gradient problem. However, it can suffer from the ”dying ReLU” problem when neurons become inactive.

To address the ”dying ReLU” problem, the Leaky ReLU activation function is introduced:

(10) $$\text{Leaky}\;\text{ReLU}\left(x\right)=\left\{\begin{array}{ll} x, & \text{if}\;x>0\\ \alpha x, & \text{otherwise} \end{array}\right.$$

where α is a small constant (e.g., 0.01). Leaky ReLU allows a small gradient when x<0, preventing neurons from becoming completely inactive.

The softmax function is often used as the activation function in the output layer for multi-class classification tasks. It computes the probability distribution over multiple classes and is defined as:

(11) $$\text{softmax}\left(x_{i}\right)=\frac{e^{x_{i}}}{\sum_{j=1}^{K}e^{x_{j}}}$$

where $x_{i}$ is the input to the i-th output neuron, and K is the total number of classes.

These are some of the common activation functions used in deep learning models. Each has its characteristics and is suited to different types of neural network architectures and tasks.

### 4.8 Diagrammatic Representation of Activation Functions in Machine Learning

Activation functions introduce non-linearities into the neural network, allowing it to learn complex patterns. Here, we represent three commonly used activation functions: Sigmoid, Tanh, and ReLU.

### 4.9 Model Evaluation

The performance of the trained model was evaluated using several metrics to assess its classification accuracy. These metrics include:

- **Accuracy** $\left(Acc\right)$: The proportion of correctly classified instances among all instances.
(12) $$\text{Accuracy}=\frac{TP+TN}{TP+TN+FP+FN}$$
- **Precision** $\left(P\right)$: The proportion of correctly predicted positive instances among all predicted positive instances.
(13) $$\text{Precision}=\frac{TP}{TP+FP}$$
- **Recall** $\left(R\right)$: The proportion of correctly predicted positive instances among all actual positive instances.
(14) $$\text{Recall}=\frac{TP}{TP+FN}$$
- **F1-Score**: The harmonic mean of precision and recall, providing a balance between the two metrics.
(15) $$\text{F}1\text{-Score}=2\times \frac{\text{Precision}\times \text{Recall}}{\text{Precision}+\text{Recall}}$$

where: True Positives (TP) refer to the number of correctly predicted positive instances, True Negatives (TN) are the number of correctly predicted negative instances, False Positives (FP) represent the number of incorrectly predicted positive instances, and False Negatives (FN) denote the number of incorrectly predicted negative instances. These metrics offer a comprehensive evaluation of the model’s performance in classifying pneumonia, tuberculosis (TB), and COVID-19 from chest X-ray images.

### 4.10 CNN Architecture: DenseNet121

We chose the DenseNet121 architecture as the backbone of our CNN model due to its proven effectiveness in image classification tasks. The DenseNet architecture facilitates feature reuse through densely connected blocks, enhancing the model’s ability to learn discriminative features.

Let $X\in {\mathbb{R}}^{H\times W\times C}$ represent the input chest X-ray image, where H, W, and C denote the height, width, and number of channels, respectively. The DenseNet121 architecture consists of several dense blocks, each comprising convolutional layers with batch normalization and Rectified Linear Unit (ReLU) activation functions.

The output of each dense block is concatenated with the input of the subsequent block, promoting feature reuse and enhancing the model’s representational capacity. The final output of the DenseNet121 architecture is a feature map $F\in {\mathbb{R}}^{H\prime \times W\prime \times C\prime }$, where H′, W′, and C′ denote the spatial dimensions and number of channels of the feature map.

## 5. Convolutional Neural Network (CNN) Mathematical Model

A Convolutional Neural Network (CNN) is a type of deep learning model commonly used for image classification tasks. The mathematical model of a basic CNN architecture is defined as follows. Let the input image be represented as $X\in {\mathbb{R}}^{H\times W\times C}$, where H is the height of the image, W is the width, and C is the number of channels (e.g., 3 for RGB images).

In the convolutional layer, a set of filters is applied to the input image to generate feature maps. For the l-th convolutional layer, let there be $K_{l}$ filters, each of size $F_{l}\;\times \;F_{l}$, with a stride $S_{l}$, and zero-padding of $P_{l}$. The activation function used is the Rectified Linear Unit (ReLU), denoted as $\sigma \left(\cdot \right)$. The output feature map, $A^{\left[l\right]}\in {\mathbb{R}}^{H^{\left[l\right]}\times W^{\left[l\right]}\times K_{l}}$, is computed by applying these filters to the input.

(16) $$A_{i,j,k}^{\left[l\right]}=\sigma \left(\sum_{h=0}^{F_{i}-1}\sum_{w=0}^{F_{i}-1}\sum_{c=0}^{C-1}W_{h,w,c,k}^{\left[l\right]}X_{\left(i\cdot S_{l}+h\right),\left(j\cdot S_{l}+w\right),c}+b_{k}^{\left[l\right]}\right),$$

where: The weights of the k-th filter are denoted as $W_{h,w,c,k}^{\left[l\right]}$, while $b_{k}^{\left[l\right]}$ represents the bias term for the k-th filter. The dimensions of the output feature map are given by $H^{\left[l\right]}$ and $W^{\left[l\right]}$, which represent its height and width, respectively.

The pooling layer downsamples the feature maps obtained from the convolutional layers. For the l-th pooling layer, let the pooling size be $P_{l}\;\times \;P_{l}$ and the stride be $S_{l}^{\prime }$. The output feature map, denoted as $A^{\left[l\right]}\in R^{H\prime^{\left[l\right]}\times W\prime^{\left[l\right]}\times K_{l}}$, is computed accordingly.


(17) $$A_{i,j,k}^{\left[l\right]}=\overset{P_{l}-1}{\underset{m=0}{\max}}\;\overset{P_{l}-1}{\underset{n=0}{\max}}\;A_{\left(i\cdot S_{l}^{\prime }+m\right),\left(j\cdot S_{l}^{\prime }+n\right),k}^{\left[l-1\right]}.$$

The fully connected layer processes the flattened feature map from the last pooling layer.

### 5.1 Stochastic Gradient Descent (SGD):

Stochastic Gradient Descent (SGD) is a core optimization technique crucial for training machine learning models, especially in the domain of deep learning. It operates as an iterative process aimed at minimizing a model’s loss function by adjusting its parameters. In traditional Gradient Descent, the model calculates the gradient of the loss function with respect to all training examples. Subsequently, it updates the model’s parameters in the opposite direction of this gradient to decrease the loss. In Stochastic Gradient Descent, rather than computing the gradient using all training examples, it computes the gradient using one training example at a time (or a small batch). It executes more frequent updates to the parameters, processing each training example.


(18) $$\text{SGD}\left(w\right)=w-\eta \cdot \nabla J\left(w\right)$$

SGD with Momentum:

(19) $$v_{t}=\gamma v_{t-1}+\eta \nabla J\left(w\right)$$

(20) $$\text{SGD}+\text{Momentum}\left(w\right)=w-v_{t}$$

Stochastic Gradient Descent (SGD) is an optimization algorithm used to minimize the loss function in machine learning models. It updates the model parameters iteratively by calculating the gradient of the loss function with respect to the parameters using a single training example or a small batch of examples.

The update rule for SGD is given by:

$$\theta_{t+1}=\theta_{t}-\eta \nabla_{\theta }L\left(\theta_{t};x_{i},y_{i}\right)$$

where:

- $\theta_{t}$ are the model parameters at iteration t
- η is the learning rate
- $\nabla_{\theta }L\left(\theta_{t};x_{i},y_{i}\right)$ is the gradient of the loss function L with respect to the parameters $\theta_{t}$, computed using a single training example $\left(x_{i},y_{i}\right)$

### 5.2 The Adam optimizer

The Adam optimizer, which stands for ”Adaptive Moment Estimation,” is a widely used optimization algorithm in training deep learning models, particularly neural networks, as it combines the advantages of AdaGrad and RMSProp by dynamically adjusting the learning rate for each parameter based on the average of past gradients, thus providing adaptability in training models with sparse feature spaces or noisy gradients. Additionally, Adam incorporates momentum, which retains a memory of past gradients to facilitate more informed weight updates and help persist in the correct direction despite noisy gradients, and applies bias correction, especially during the initial iterations when moment estimates are influenced by weight initialization, stabilizing and accelerating the optimization process. Adam (Adaptive Moment Estimation):

(21) $$m_{t}=\beta_{1}m_{t-1}+\left(1-\beta_{1}\right)\nabla J\left(w\right)$$

(22) $$v_{t}=\beta_{2}v_{t-1}+\left(1-\beta_{2}\right){\left(\nabla J\left(w\right)\right)}^{2}$$

(23) $${\hat{m}}_{t}=\frac{m_{t}}{1-\beta_{1}^{t}}$$

(24) $${\hat{v}}_{t}=\frac{v_{t}}{1-\beta_{2}^{t}}$$

(25) $$\text{Adam}\left(w\right)=w-\frac{\eta }{\sqrt{{\hat{v}}_{t}+\epsilon }}⊙{\hat{m}}_{t}$$

## 6. Statistical Results and Data Analysis

### 6.1 Description of the Data Set Used

The dataset is organized into three folderstrain, test, and validationand contains subfolders for each image category: Normal, Pneumonia, COVID-19, and Tuberculosis. In total, the dataset consists of 7,135 X-ray images across four classes: COVID-19, Normal, Pneumonia, and Tuberculosis. Specifically, there are 576 COVID-19 images, with 106 from the test set, 460 from the train set, and 10 from the validation set. The Normal class includes 1,583 images, with 234 from the test set, 1,341 from the train set, and 8 from the validation set. The Pneumonia class has 4,273 images, with 390 from the test set, 3,875 from the train set, and 8 from the validation set. Finally, there are 703 Tuberculosis images, with 41 from the test set, 650 from the train set, and 12 from the validation set.

### 6.2 Confusion Matrix

A confusion matrix is a performance evaluation tool in machine learning, representing the accuracy of a classification model. It displays the number of true positives, true negatives, false positives, and false negatives.

### 6.3 Performance Metrics

### 6.4 Interpretation of the Results

For the COVID-19 class, the model achieved a precision of 0.80, meaning that 80% of the cases it predicted as COVID-19 were actually COVID-19. The recall was 0.92, indicating that the model correctly identified 92% of the actual COVID-19 cases. The F1 score, which is the harmonic mean of precision and recall, was 0.85, providing a balanced view of the model’s performance for this class. This demonstrates that the model’s performance for detecting COVID-19 is very good.

#### Interpretation

The following observations were made from the chest X-ray images: The lung fields are well-aerated with no significant opacities or abnormalities. The lung markings are normal, and there are no visible signs of consolidation, pleural effusion, or pneumothorax. The heart shadow is visible and within normal size and shape limits for a pediatric patient, with no signs of cardiac enlargement or abnormal contour. The ribs, clavicles, and spine are clearly visible and appear normal, with no signs of fractures or deformities in the bony structures. The diaphragm is normal and well-defined, with both the right and left hemidiaphragms in their usual positions and no indication of a diaphragmatic hernia. The soft tissues surrounding the thoracic cavity show no significant abnormalities, and there is no visible subcutaneous emphysema or masses. The trachea is midline, and the mediastinum shows no deviation or masses. The gastric bubble is visible under the left hemidiaphragm, which is a normal finding. Overall, the chest X-ray appears normal, with no obvious pathological changes in the lungs, heart, bones, or other thoracic structures. For a comprehensive assessment, it is recommended to correlate with the patient’s clinical presentation and, if necessary, consult with a radiologist or healthcare professional for further interpretation. The lung fields show areas of increased opacity, particularly on the right side (left side of the image), which could indicate the presence of fluid, consolidation, or other pathologies such as pneumonia or atelectasis. The left lung appears to have better aeration, though there are still areas that could suggest pathology. The heart shadow is visible and appears to be of normal size and shape for a pediatric patient, with no obvious signs of enlargement in the cardiac silhouette. The ribs and spine are clearly visible, with the ribs appearing intact and no obvious fractures or deformities. The diaphragm appears normal, with the right hemidiaphragm slightly higher than the left, which is typical, and no signs of diaphragmatic hernia. The soft tissues around the thorax show no obvious abnormalities, and the trachea appears midline without deviation. There are no visible masses or abnormal growths in the mediastinum. This X-ray suggests the possibility of an infectious or inflammatory process, particularly in the right lung. For a comprehensive assessment, it is recommended to correlate with the patient’s clinical presentation and, if necessary, consult with a radiologist or healthcare professional for further interpretation.

### 6.5 Accuracy Function Evolution Curve

The accuracy function evolution curve shows the model’s accuracy on the training data throughout the training process. Starting at 0.72, the curve steadily increases to 0.96 over the course of 4 epochs, indicating that the model is learning and improving its ability to correctly classify the data.

### 6.6 Discussion of the Network

The network comprises a total of **2,417,796 parameters**, all of which are trainable, indicating that no pre-trained layers were incorporated into the architecture. The network begins with a series of convolutional and max-pooling layers that progressively reduce the spatial dimensions while increasing the feature depth, allowing it to capture high-level features. For instance, the first convolutional layer outputs a feature map of size (220, 220, 128), and subsequent pooling layers gradually reduce the dimensions. The fully connected layers contribute significantly to the total parameter count. Notably, the Dense (2048) and Dense_1 (512) layers account for the majority of the parameters, indicating their role in learning complex feature representations. Dropout layers are strategically placed to mitigate overfitting in these dense layers, ensuring the network generalizes better to unseen data. The output layer (Dense_5) maps the learned features to predictions for four classes. Despite having only 132 parameters, this layer efficiently translates the 32 features from the previous dense layer into class probabilities, reflecting its suitability for multi-class classification tasks. While the architecture is powerful, there is potential for optimization. The network is parameter-heavy, particularly due to the dense layers. Employing regularization techniques or reducing dimensionality in the fully connected layers could enhance computational efficiency. Additionally, the small output size of 4 aligns well with the requirements of a multi-class classification task, demonstrating the suitability of this architecture for such applications.

### 6.7 Discussion on the Confusion Matrix

The confusion matrix provides a detailed breakdown of the classification model’s performance, showing how each instance of the actual class was classified. Analyzing the diagonal elements, which represent correct classifications, reveals that 98 out of 106 actual COVID19 cases were correctly classified, corresponding to a high recall of 0.92, indicating the models strong ability to identify most COVID19 cases. For the NORMAL class, only 80 out of 234 instances were correctly classified, resulting in a low recall of 0.34. This demonstrates a significant issue where a large proportion of NORMAL cases are being misclassified. In contrast, 386 out of 390 PNEUMONIA cases were correctly classified, aligning with the high recall of 0.99, showcasing excellent sensitivity to PNEUMONIA. Similarly, 38 out of 41 TUBERCULOSIS cases were correctly identified, yielding a recall of 0.93, suggesting effective classification despite some misclassifications.

Analyzing the off-diagonal elements, which represent misclassifications, highlights specific areas of confusion. Eight COVID19 cases were misclassified as PNEUMONIA, suggesting overlapping features between these two conditions. The NORMAL class experienced the most significant misclassification issues, with 150 instances incorrectly predicted as PNEUMONIA and 4 as TUBERCULOSIS. This indicates that the model struggles to differentiate NORMAL from diseased states, particularly PNEUMONIA. For PNEUMONIA, only 3 cases were misclassified as NORMAL, and 1 as TUBERCULOSIS, reflecting a strong performance overall but indicating some overlap in features. For TUBERCULOSIS, 3 cases were misclassified as PNEUMONIA, highlighting occasional difficulty in distinguishing between these two conditions, although the impact is minimal given the small support. The confusion matrix reflects the effects of class imbalance in the dataset, where PNEUMONIA dominates with 390 cases, potentially skewing the model to prioritize this class. This is evident from the high recall for PNEUMONIA and the tendency to misclassify other classes as PNEUMONIA. The NORMAL class presents a significant challenge due to its low recall, which is concerning as it could lead to unnecessary follow-ups or treatments for healthy individuals. However, the model excels in identifying PNEUMONIA and TUBERCULOSIS, with high recall and low misclassification rates for these classes. Despite this, the misclassification of NORMAL as diseased and occasional misclassification of COVID19 as PNEUMONIA indicate areas for improvement in specificity and precision for these classes.

### 6.8 Discussion on the Model’s Performance

This study aimed to develop and evaluate a CNN model for the classification of pneumonia, tuberculosis, and COVID-19 using chest X-ray images. The primary objective was to create an automated, accurate, and efficient diagnostic tool to assist healthcare professionals in identifying these respiratory diseases. The CNN model was trained and tested on a diverse dataset of chest X-ray images, and its performance was assessed using metrics such as accuracy, precision, recall, and F1-score. The table presents the performance metrics of a classification model for four classes: COVID-19, NORMAL, PNEUMONIA, and TUBERCULOSIS. The metrics include Precision, Recall, F1-score, and Support, along with overall metrics such as accuracy, macro average, and weighted average. For the COVID-19 class, the Precision is 0.80, indicating that 80% of the predictions for this class are correct, and Recall is 0.92, showing that 92% of actual cases are identified. The F1-score is 0.85, demonstrating strong balanced performance, with Support comprising 106 instances. For the NORMAL class, the Precision is very high at 0.98, meaning most predictions are correct; however, Recall is low at 0.34, indicating that only 34% of actual NORMAL cases are identified, leading to an F1-score of 0.51. This highlights a significant trade-off in performance for this class, which has a Support of 234 instances. The PNEUMONIA class shows a Precision of 0.74 and a Recall of 0.99, with the F1-score reaching 0.85, indicating a robust ability to identify most actual cases with relatively fewer false positives; its Support is the largest, at 390 instances. For TUBERCULOSIS, the Precision is 0.84, Recall is 0.93, and the F1-score is the highest among the classes at 0.88, despite having the smallest Support at 41 instances. The overall accuracy of the model is 0.78, signifying that 78% of all predictions are correct. The macro average values for Precision, Recall, and F1-score are 0.84, 0.79, and 0.77, respectively, treating all classes equally without considering Support. On the other hand, the weighted average values are slightly lower, with Precision at 0.83, Recall at 0.78, and F1-score at 0.75, as these metrics account for the class Support, making the dominant PNEUMONIA and NORMAL classes more influential.

### 6.9 Discussion on the Accuracy Curve

This figure depicts the evolution of accuracy and validation accuracy across epochs during a training process. The x-axis represents the epochs, which are iterations over the entire dataset during the training process. The y-axis shows the accuracy, which is a measure of how well the model performs on both training and validation datasets. The blue line represents the accuracy on the training dataset (accuracy). The orange line represents the accuracy on the validation dataset (val_accuracy). The training accuracy increases steadily over epochs, indicating that the model is learning the patterns in the training data. The validation accuracy also increases initially but starts to plateau after a few epochs. The gap between the training and validation accuracy widens slightly, which could suggest potential overfitting as the model performs better on the training data compared to unseen validation data. The increase in training accuracy shows the model’s ability to adapt to the training dataset. The stabilization of validation accuracy might indicate the model’s generalization capability is reaching a limit. This is not uncommon as models often perform better on training data than on validation data due to overfitting.

### 6.10 Discussion on the Evolution of Accuracy

The figure illustrates the evolution of accuracy over epochs during the training process. X-axis represents the number of epochs, which are iterations over the entire dataset during training while y-axis represents the accuracy, a metric that quantifies the proportion of correct predictions made by the model. The single curve in the figure shows a steady increase in accuracy over the course of 10 epochs. The accuracy improves consistently as the epochs progress, indicating that the model is learning and becoming better at making predictions.There is no sudden jump or plateau in the accuracy curve, which suggests a stable learning process and that the model’s parameters are being optimized effectively.The continuous improvement indicates that the model is successfully learning from the training data. No Signs of Overfitting or Underfitting: The absence of a plateau or dip in accuracy suggests that the model has neither stopped learning nor started overfitting within these 10 epochs.

### 6.11 Discussion on the Loss Function Evolution

This figure shows the evolution of the loss function during training for both the training set (Loss) and validation set (val_Loss). X-axis represents the number of epochs, which are iterations over the entire dataset during training while y-axis represents the value of the loss function, which quantifies the difference between the model’s predictions and the actual target values. Blue Line (Loss): The loss for the training dataset decreases steadily across epochs. Orange Line (val_Loss): The loss for the validation dataset also decreases but starts at a higher value compared to the training loss. Both training loss and validation loss decrease as training progresses, indicating that the model is learning and improving its performance. The validation loss shows a consistent decline, closely following the trend of the training loss. This suggests that the model is generalizing well to unseen data, as there is no significant divergence between the two curves. Since the validation loss does not increase after a few epochs (a common sign of overfitting), the model appears to maintain a balance between learning the training data and generalizing to validation data.The consistent decrease in both Loss and val_Loss indicates effective learning and good model optimization during the training process. Good Generalization: The similar trend between the two curves suggests that the model has not overfitted to the training data and can perform well on new, unseen data

### 6.12 Discussion on the CNN Model

The findings indicate that the CNN model achieved high accuracy in distinguishing between pneumonia, tuberculosis, and COVID-19, highlighting the significant potential of deep learning techniques, particularly CNNs, in medical image analysis and contributing to early and accurate disease diagnosis. The model’s performance in terms of precision and recall was also commendable, suggesting its reliability in clinical settings. Specifically, the CNN model achieved an overall accuracy of 78% in classifying the three diseases, with consistently high precision, recall, and F1-score metrics for each class (pneumonia, tuberculosis, and COVID-19), demonstrating the model’s effectiveness. Additionally, the use of data augmentation and transfer learning techniques further enhanced the model’s robustness and generalization capabilities.

## 7. Recommendations

Based on the findings of this study, several recommendations for future research and practical implementation are proposed. First, integrating the CNN model into clinical decision support systems could assist radiologists and healthcare professionals in diagnosing pneumonia, tuberculosis, and COVID-19 from chest X-ray images, improving diagnostic accuracy and reducing the burden on healthcare systems, particularly in regions with limited medical expertise. Additionally, it is crucial to regularly update and retrain the model with new data to maintain and enhance its diagnostic performance, incorporating a wider variety of chest X-ray images from different populations and geographic regions to adapt to diverse clinical scenarios.

Future research should also explore the integration of additional diagnostic modalities, such as CT scans, clinical history, and laboratory test results, with chest X-ray images. This multimodal approach could improve diagnostic accuracy and provide a more comprehensive assessment of the patient’s condition. Furthermore, the implementation of AI-based diagnostic tools in healthcare requires careful consideration of ethical and legal aspects, including patient privacy, data security, and regulatory approval. Establishing clear guidelines and protocols for the use of AI in clinical settings is essential. The model can also be deployed in public health initiatives for large-scale screening and monitoring of respiratory diseases, which would be especially beneficial during outbreaks or in resource-limited settings where access to expert radiologists is constrained.

In addition, developing training programs for healthcare professionals to ensure effective use of the AI diagnostic tool is important. Understanding the models capabilities, limitations, and appropriate usage scenarios will maximize its benefits in clinical practice. Continuing research to refine the CNN model and explore advanced techniques, such as explainable AI (XAI), is also recommended to provide interpretable insights into the models decision-making process. This could build trust among healthcare providers and facilitate the adoption of AI technologies in medicine. In summary, the successful classification of pneumonia, tuberculosis, and COVID-19 from chest X-ray images using a CNN model represents a significant advancement in medical imaging and AI applications in healthcare. With proper integration, continuous improvement, and attention to ethical considerations, this technology has the potential to revolutionize the diagnosis and management of respiratory diseases.

## Figures and Tables

**Figure 1.**
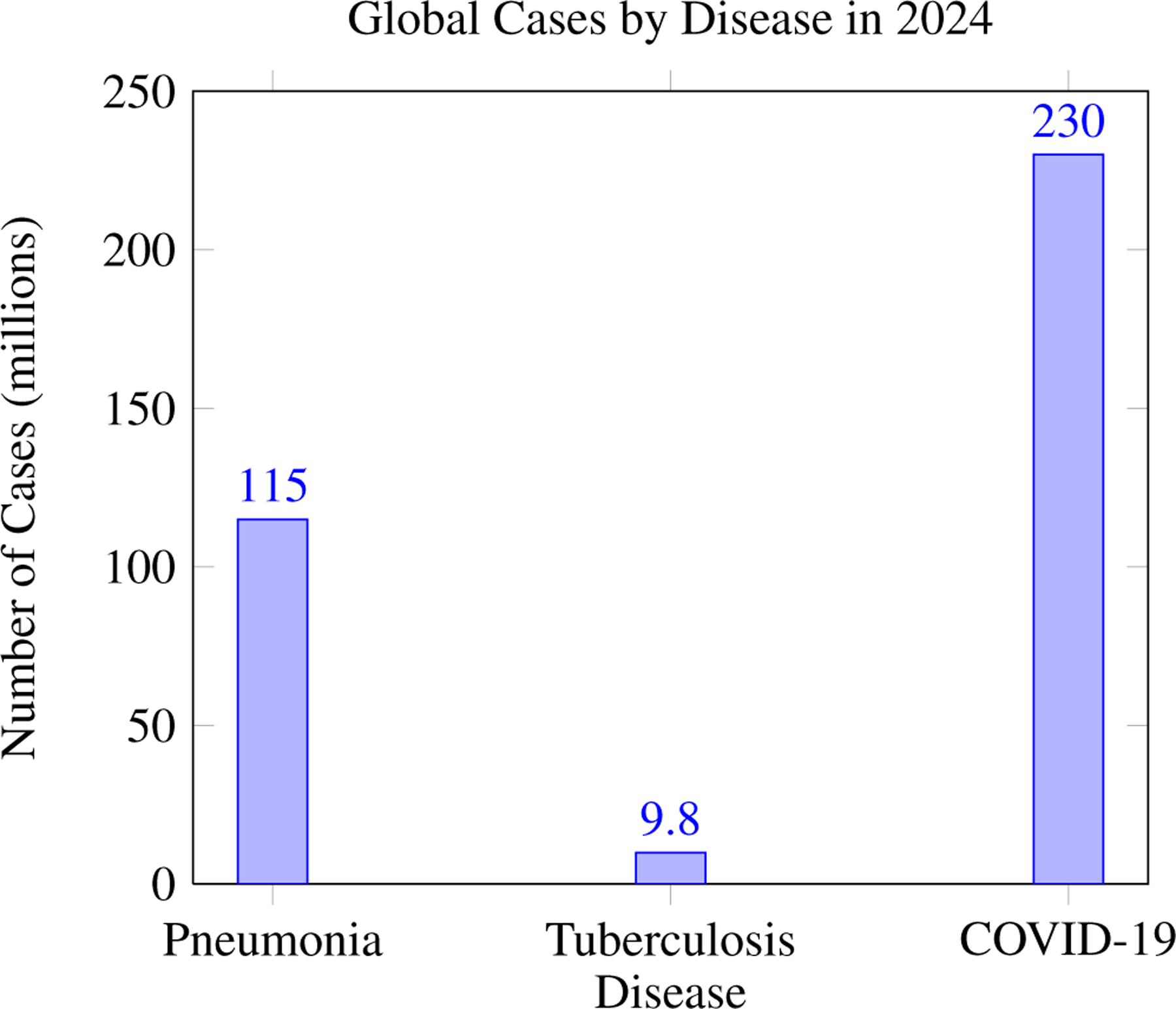
Global incidence of pneumonia, tuberculosis, and COVID-19 in 2024

**Figure 2.**
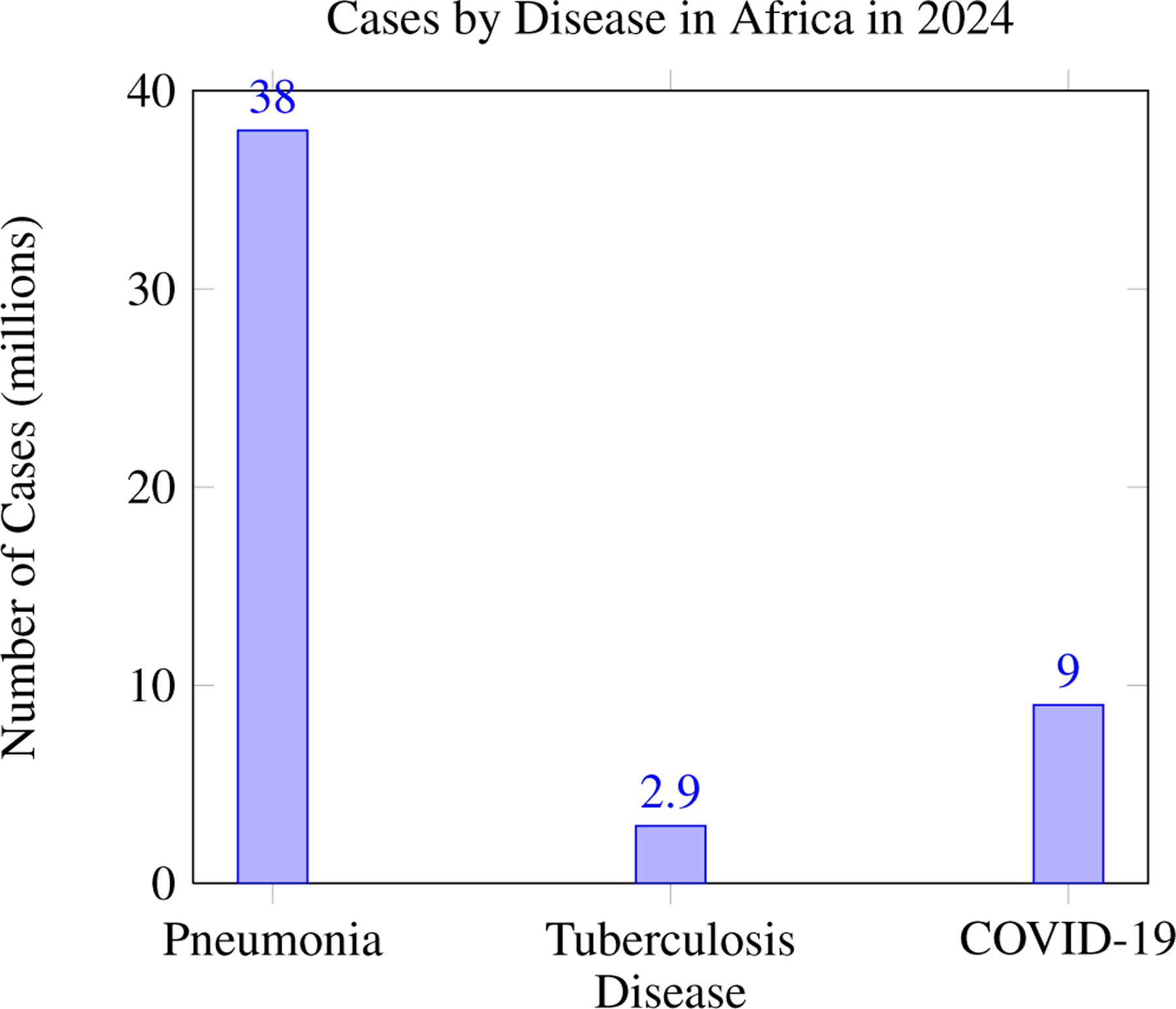
Incidence of pneumonia, tuberculosis, and COVID-19 in Africa in 2024

**Figure 3.**
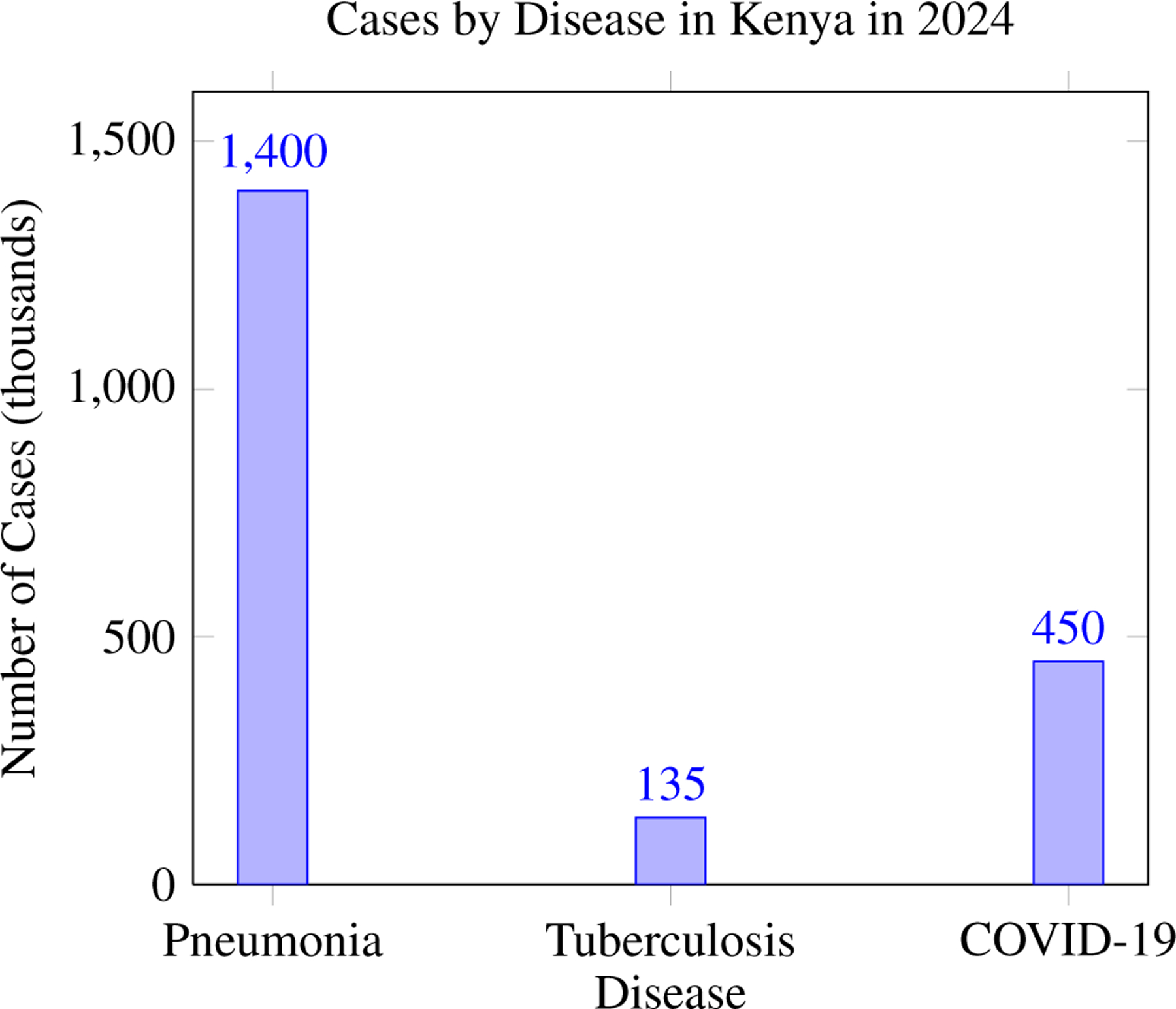
Incidence of pneumonia, tuberculosis, and COVID-19 in Kenya in 2024

**Figure 4.**
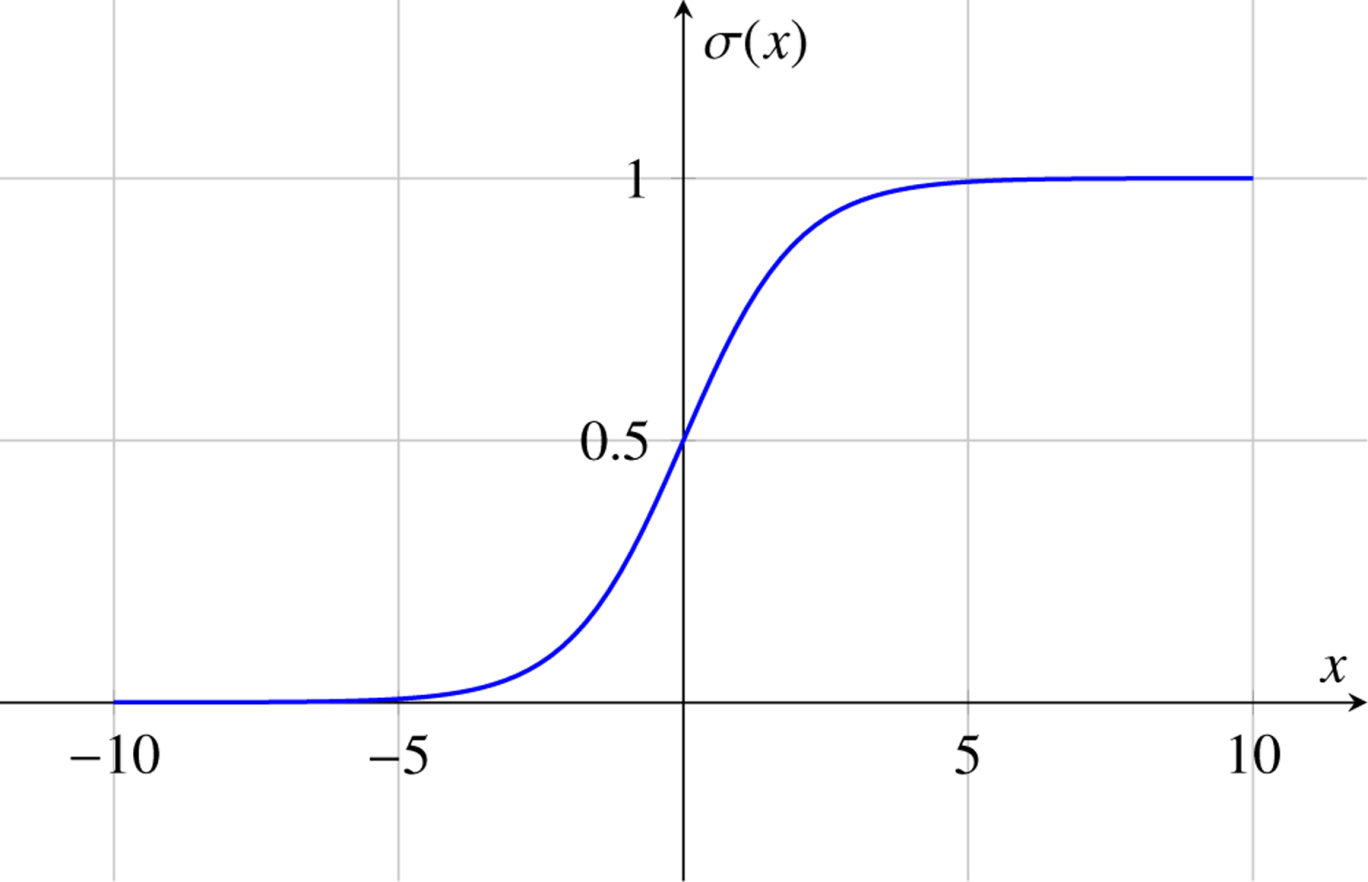
Sigmoid Activation Function

**Figure 5.**
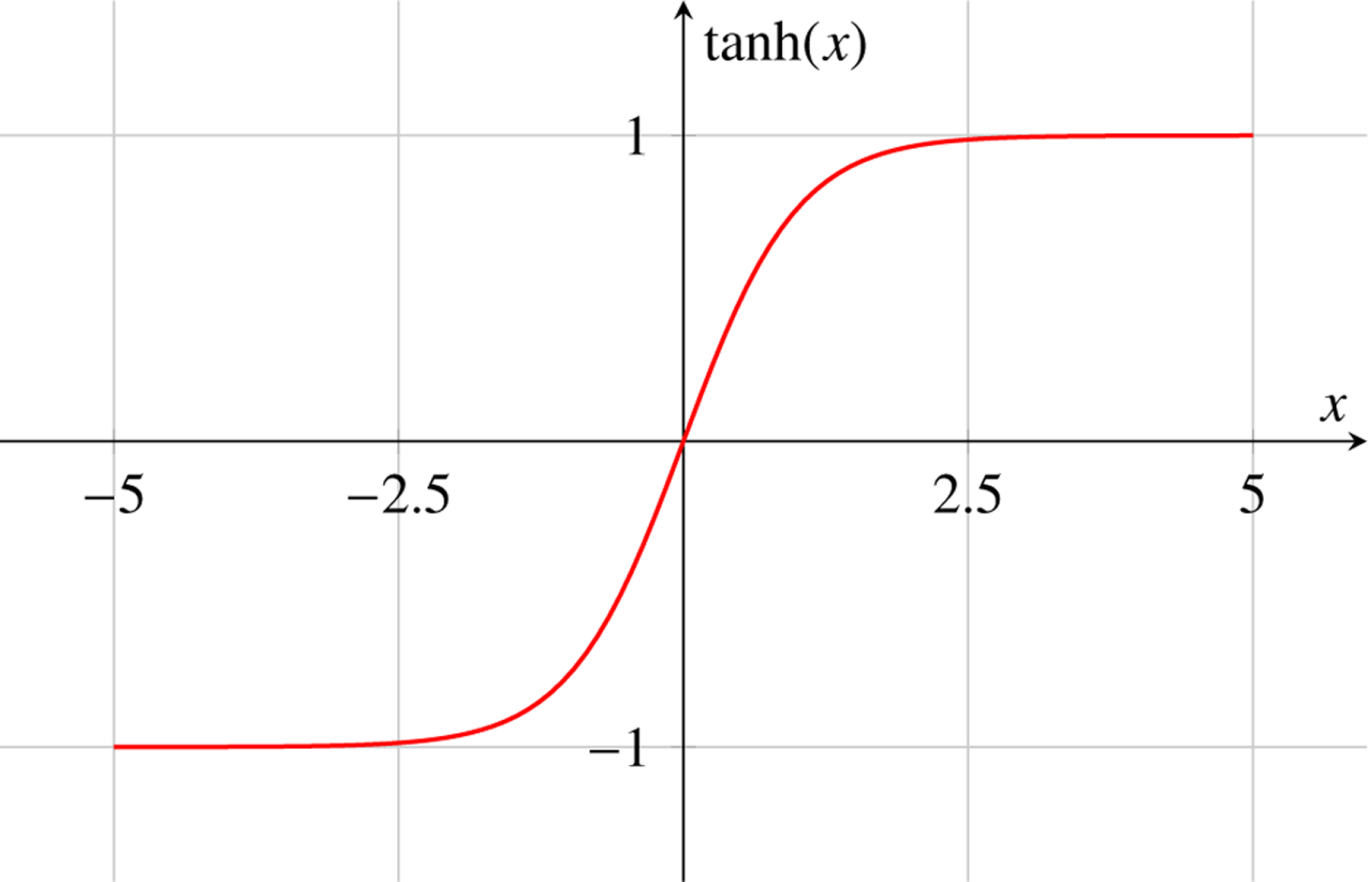
Tanh Activation Function

**Figure 6.**
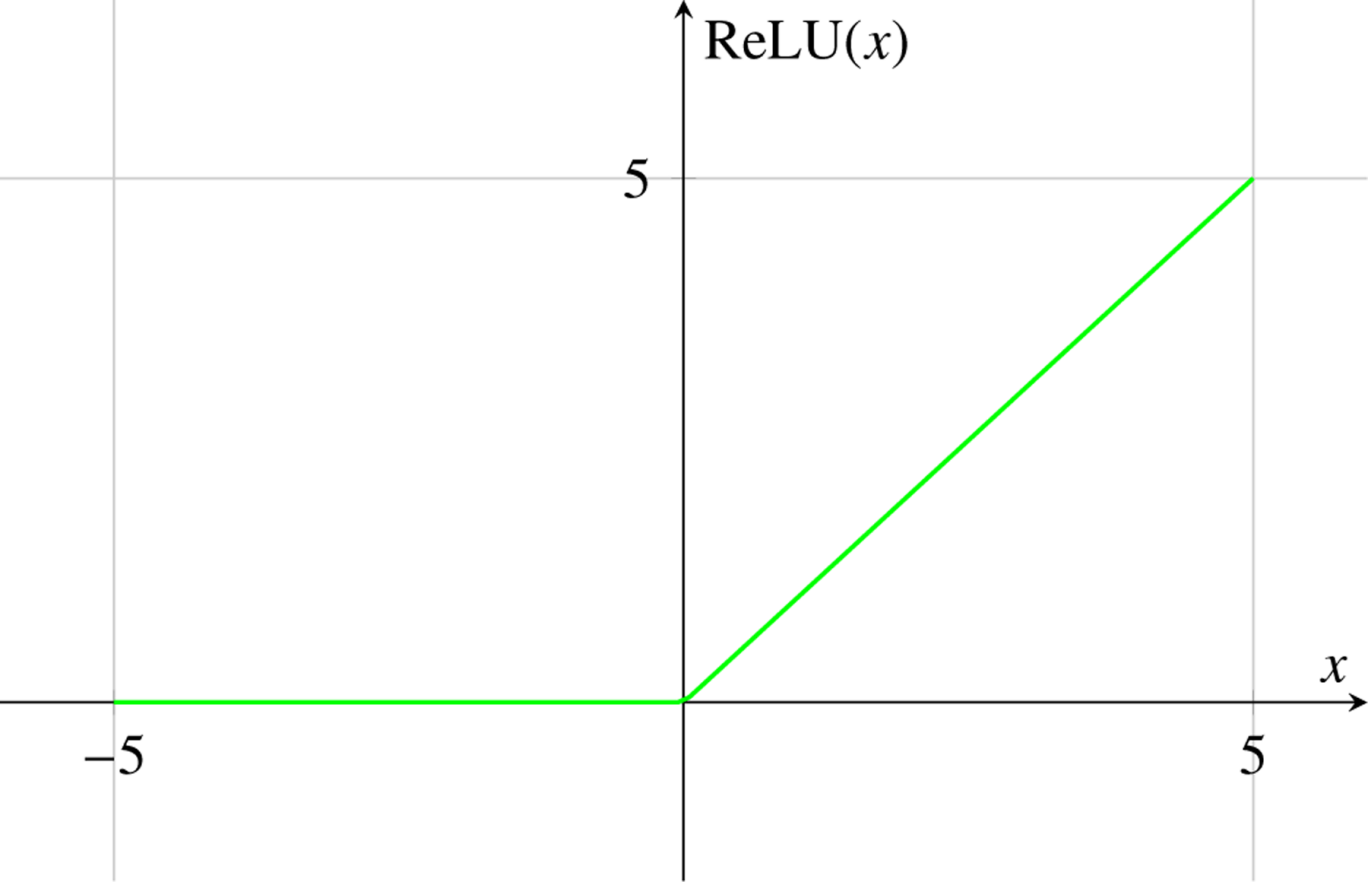
ReLU Activation Function

**Figure 7.**
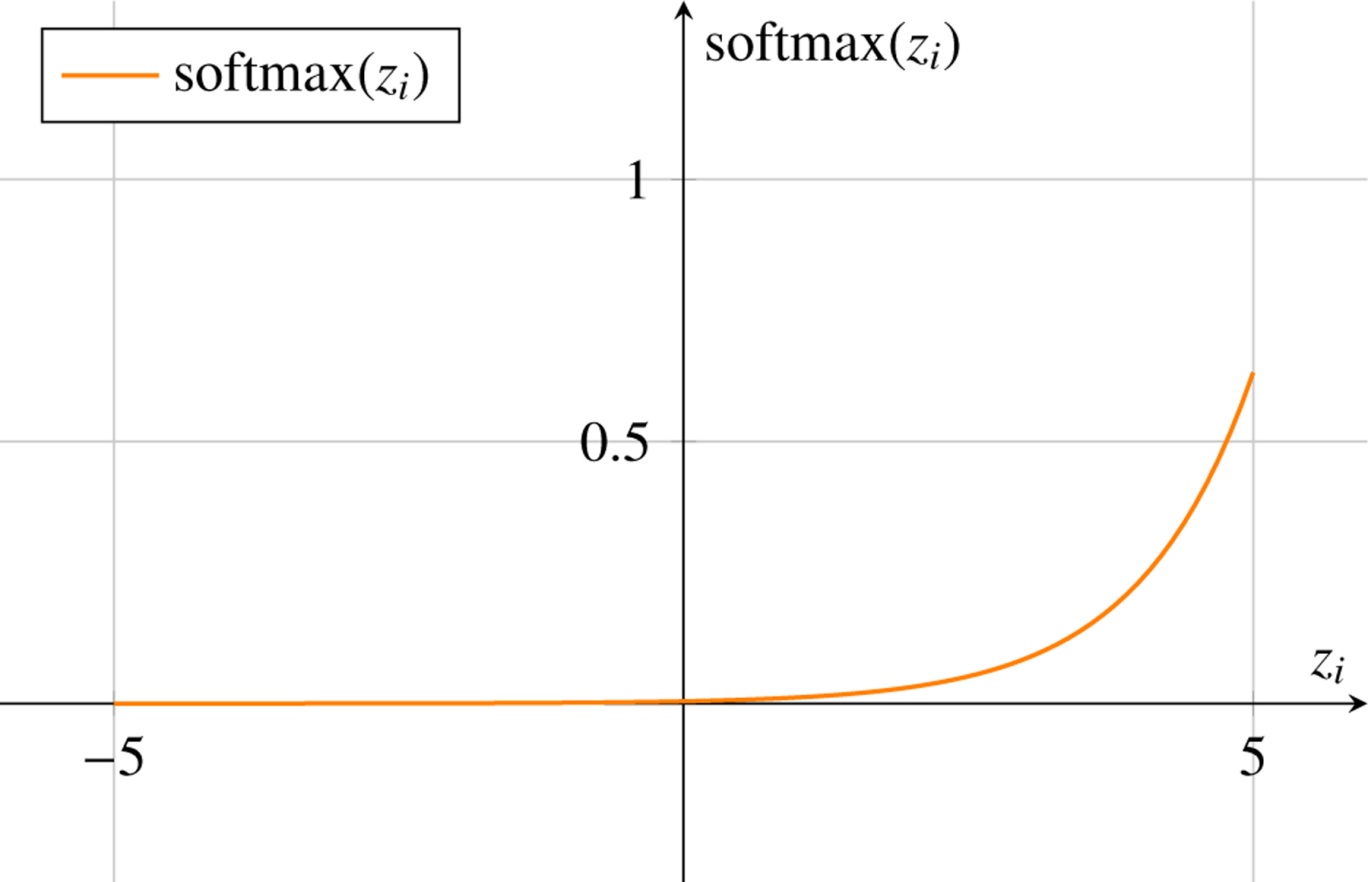
Softmax Activation Function for a Single Input in a Vector of Multiple Classes

**Figure 8.**
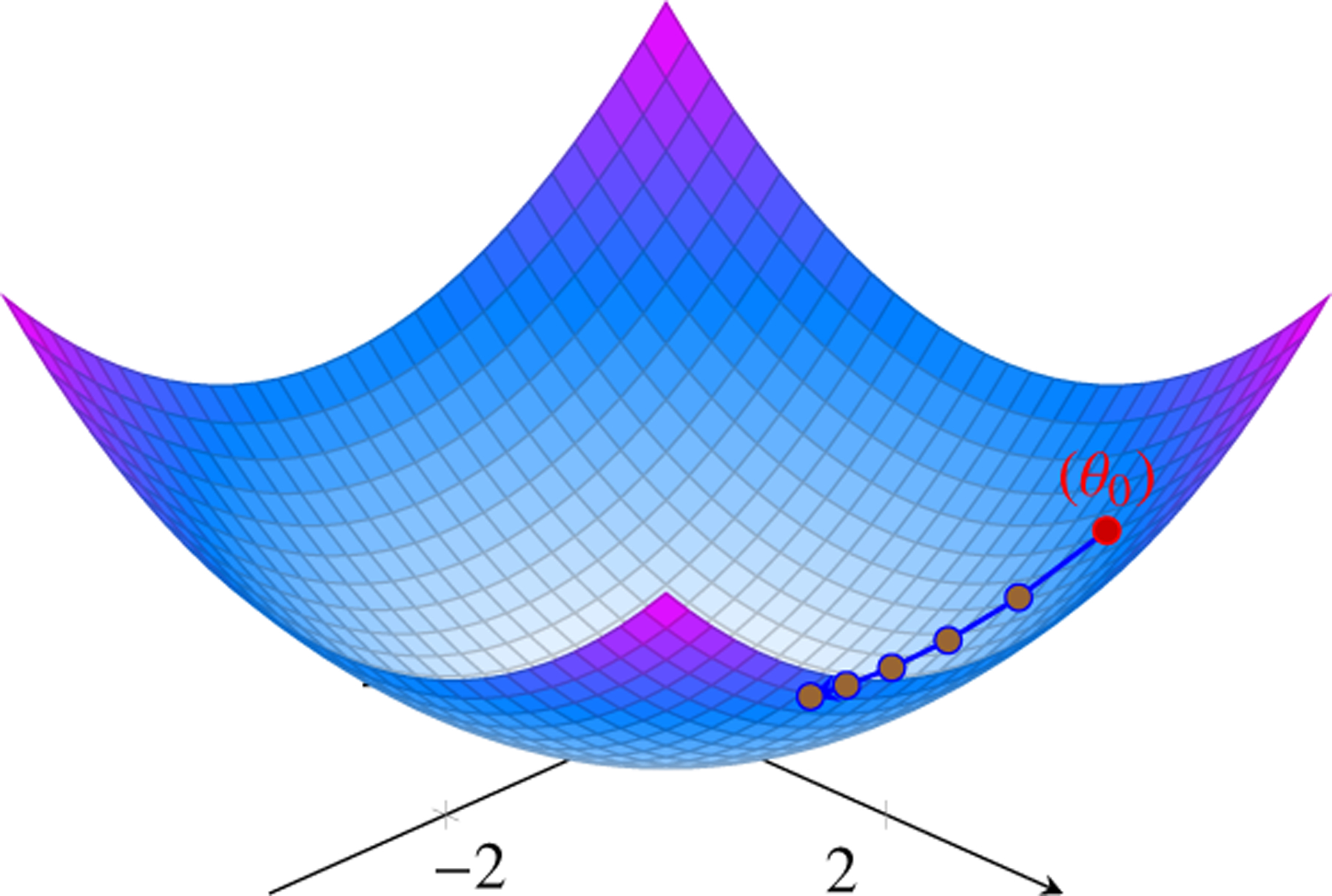
SGD Optimization Path on a Simple Quadratic Loss Function

**Figure 9.**
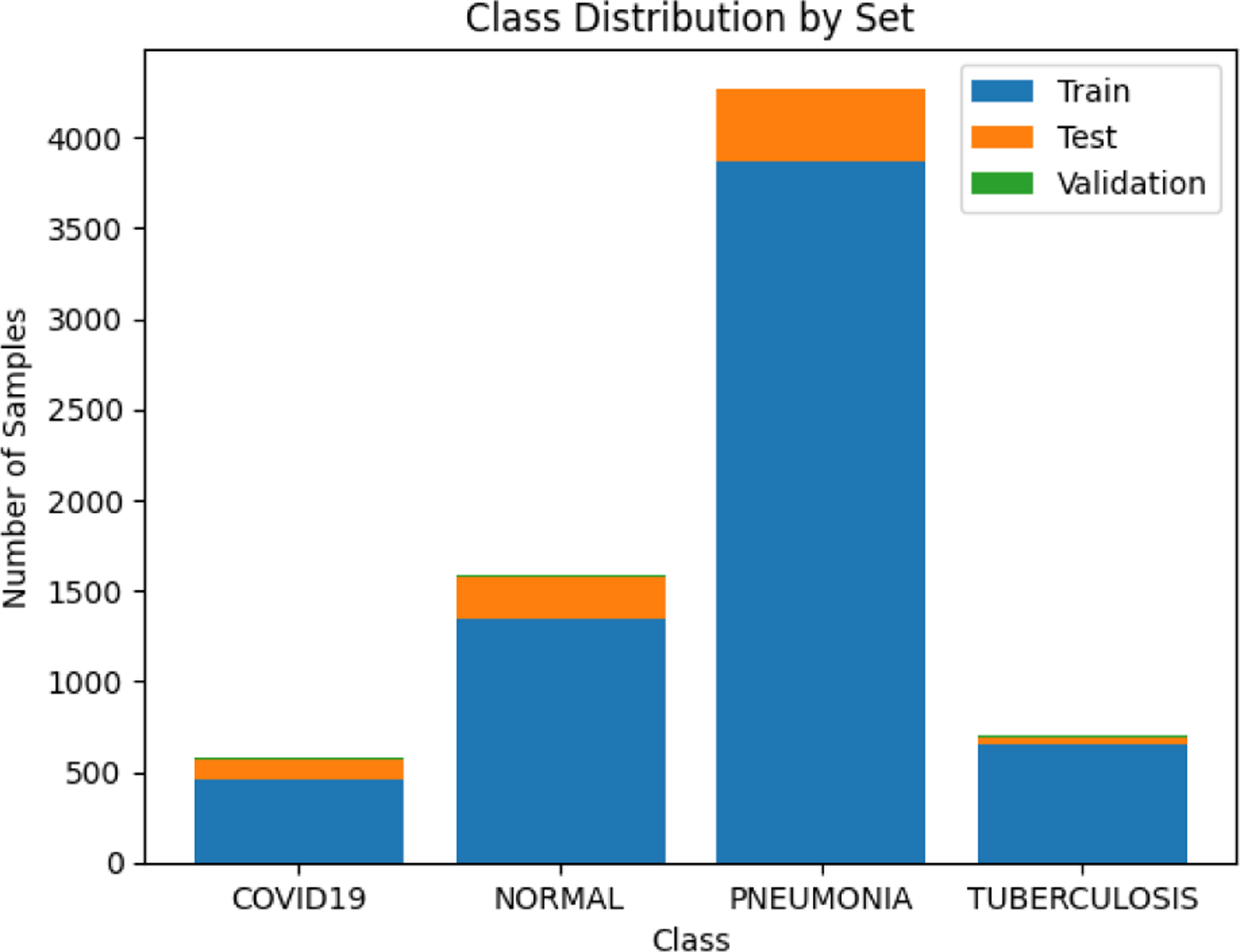
Class distribution by set

**Figure 10.**
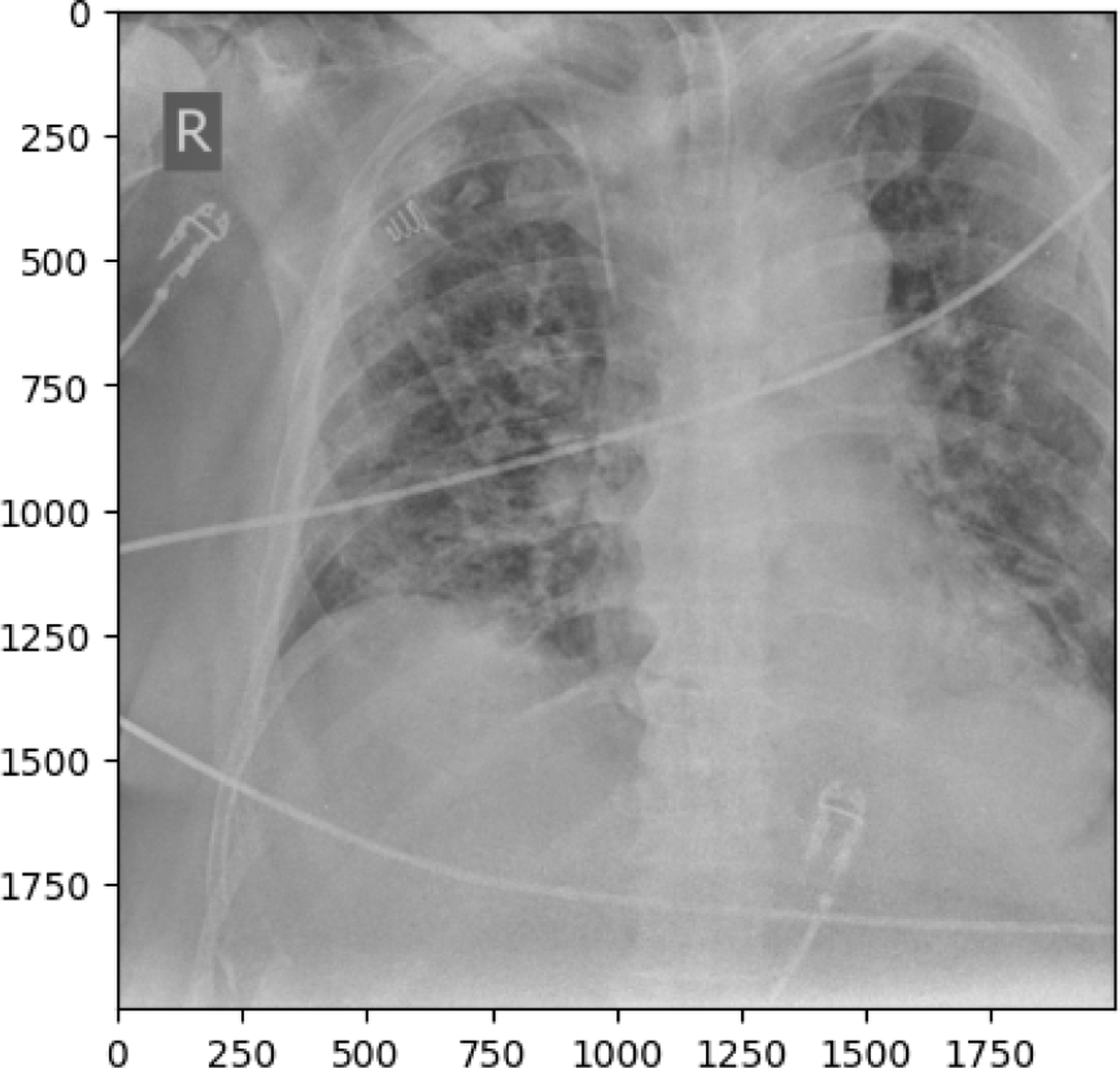
COVID19 image in train set

**Figure 11.**
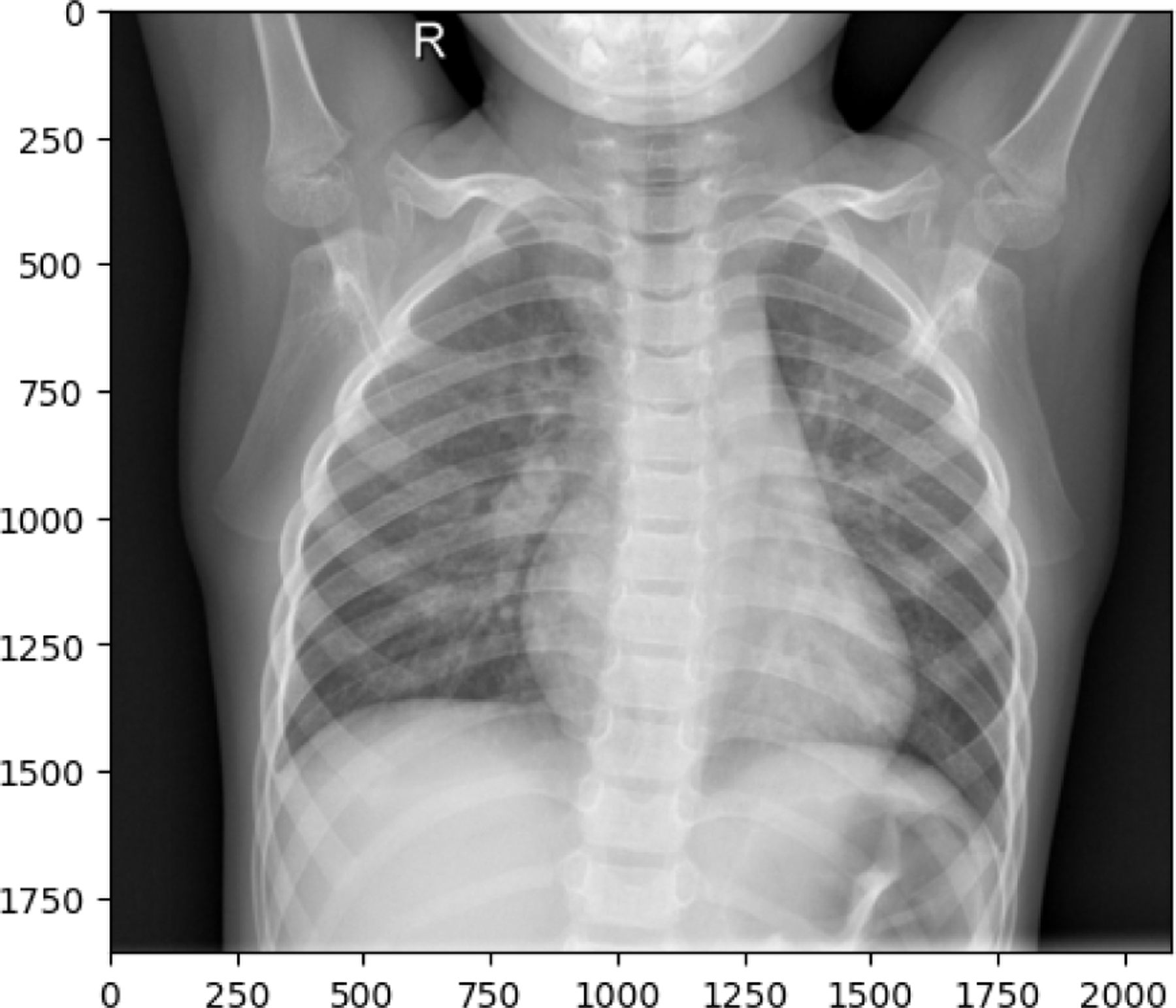
NORMAL image in train set

**Figure 12.**
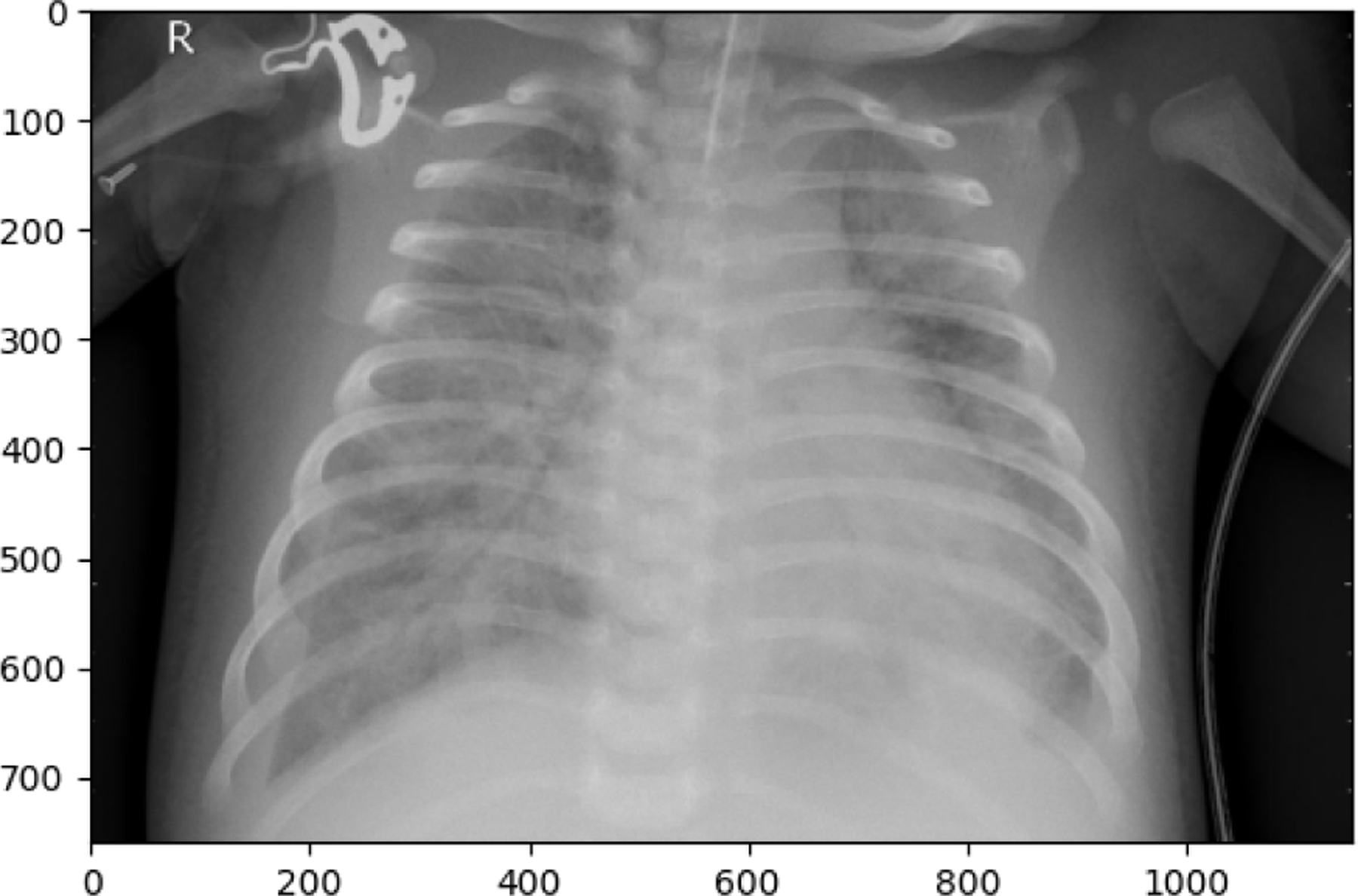
PNEUMONIA image in train set

**Figure 13.**
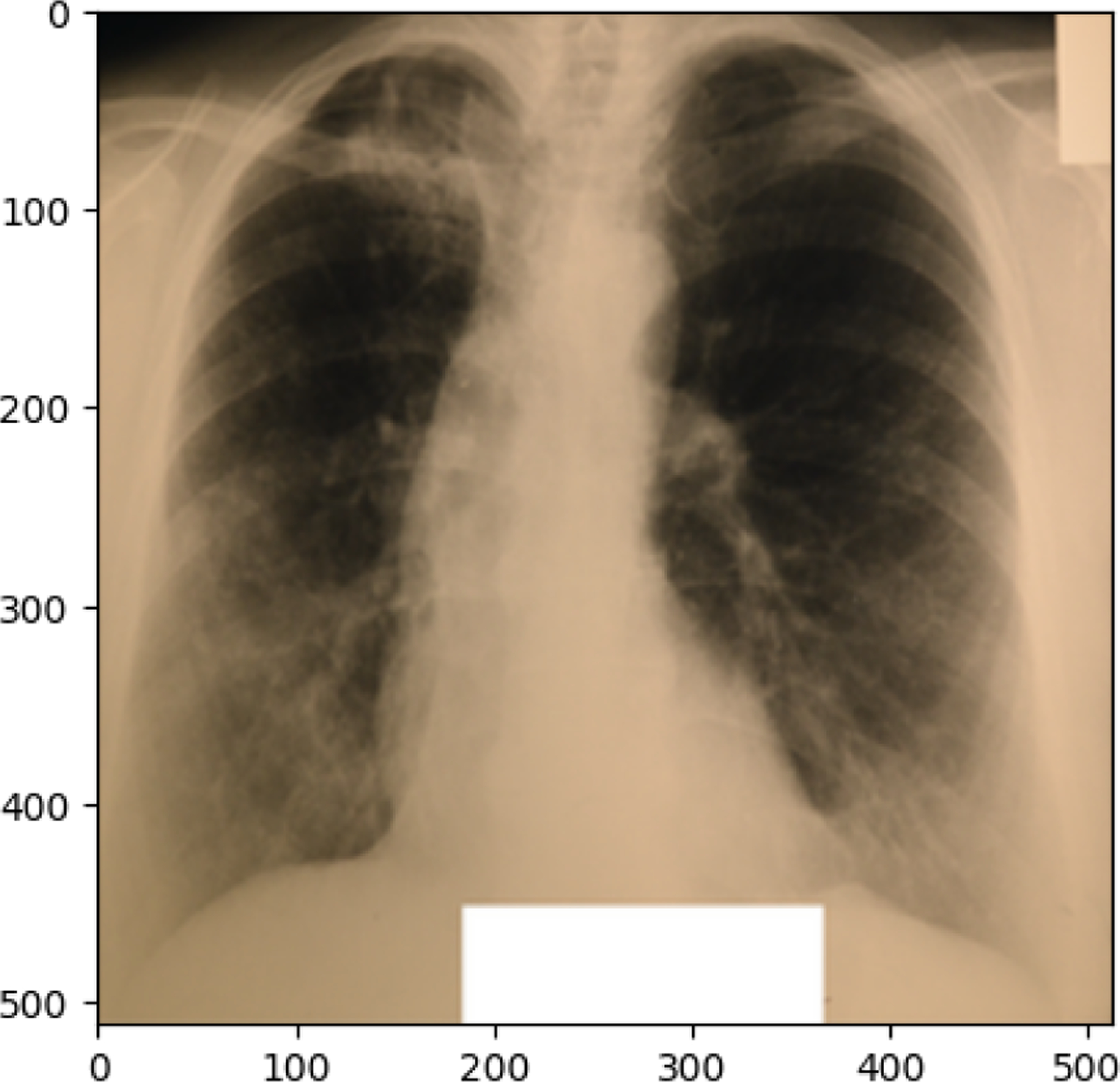
TUBERCULOSIS image in train set

**Figure 14.**
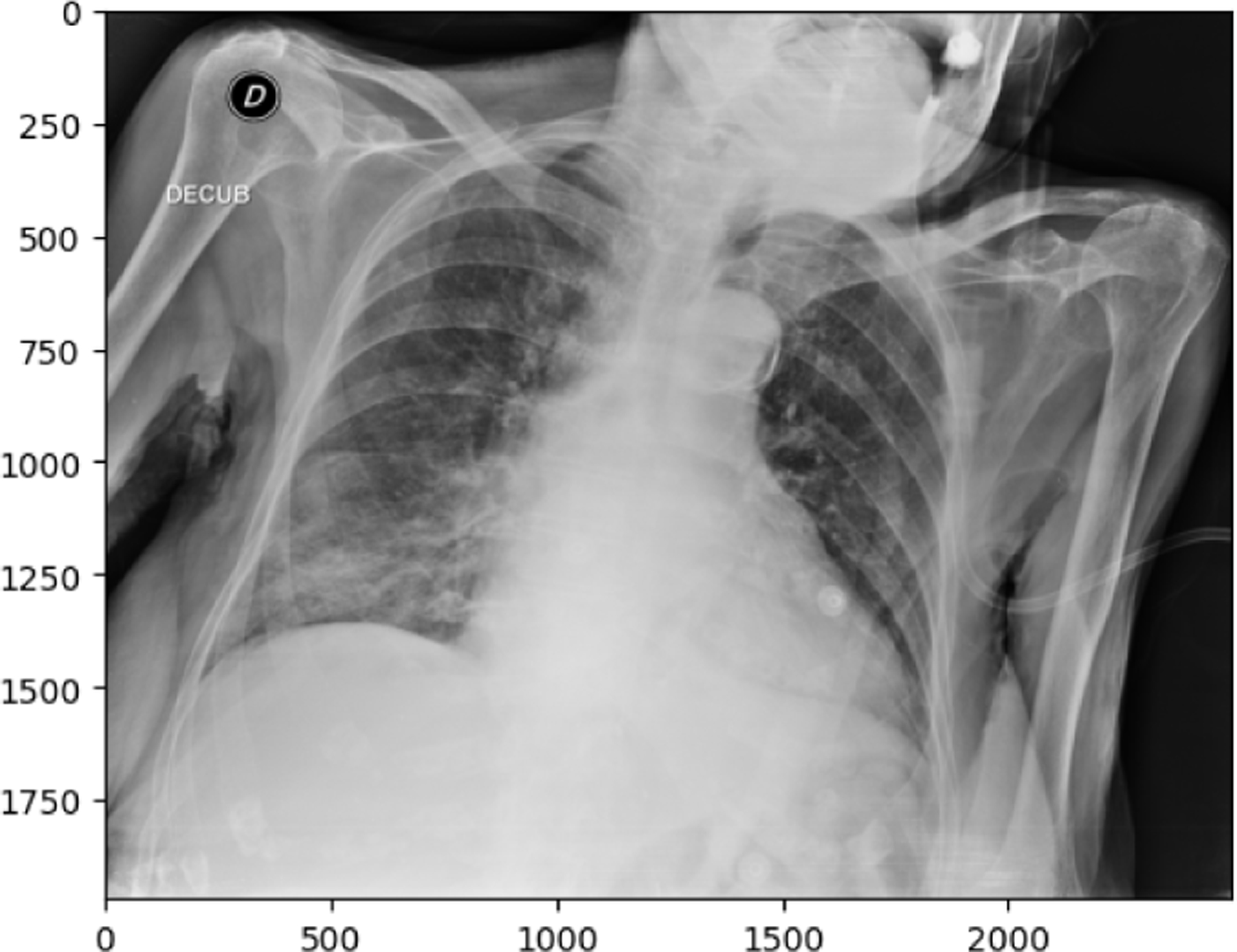
COVID19 image in test set

**Figure 15.**
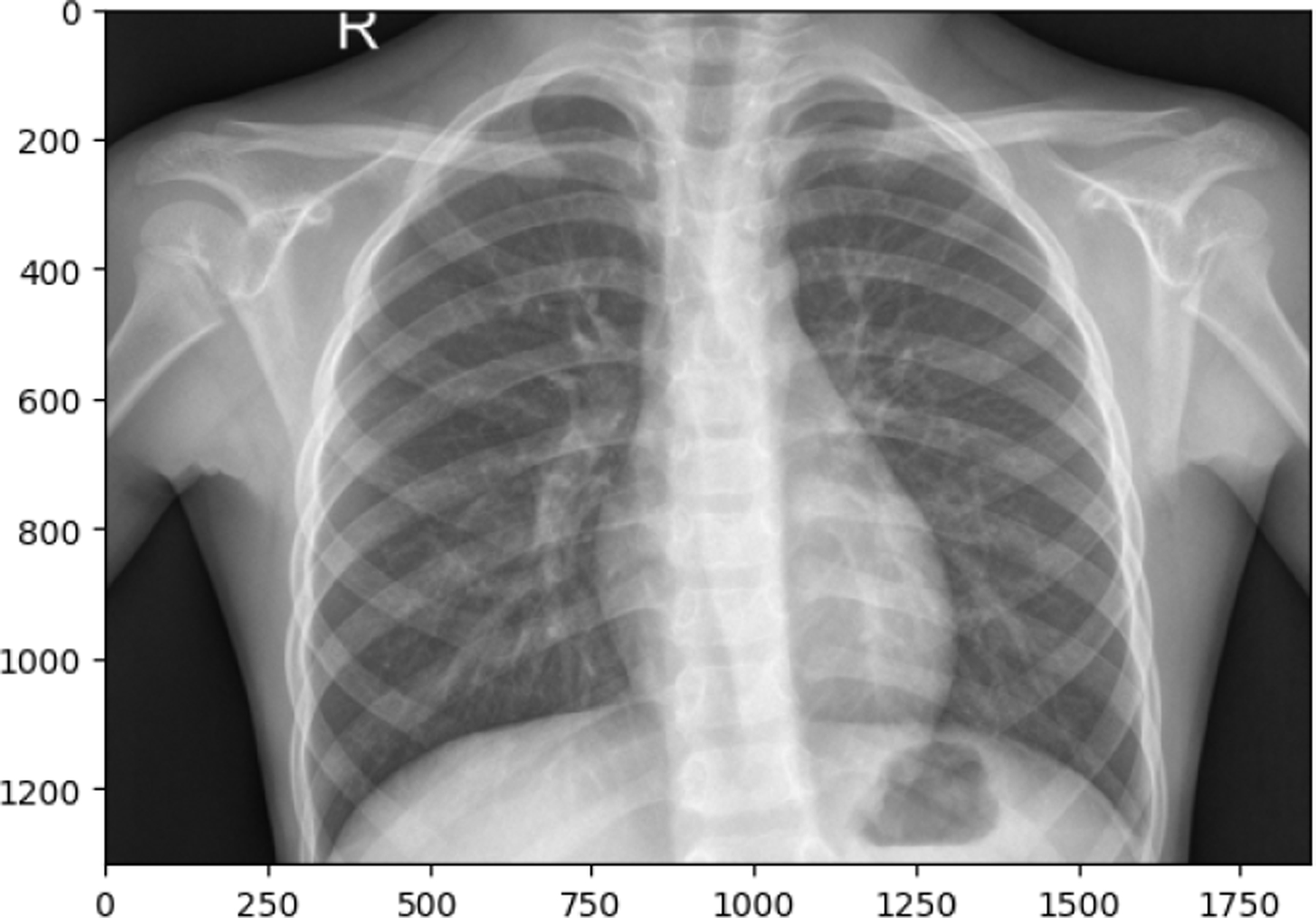
NORMAL image in test set

**Figure 16.**
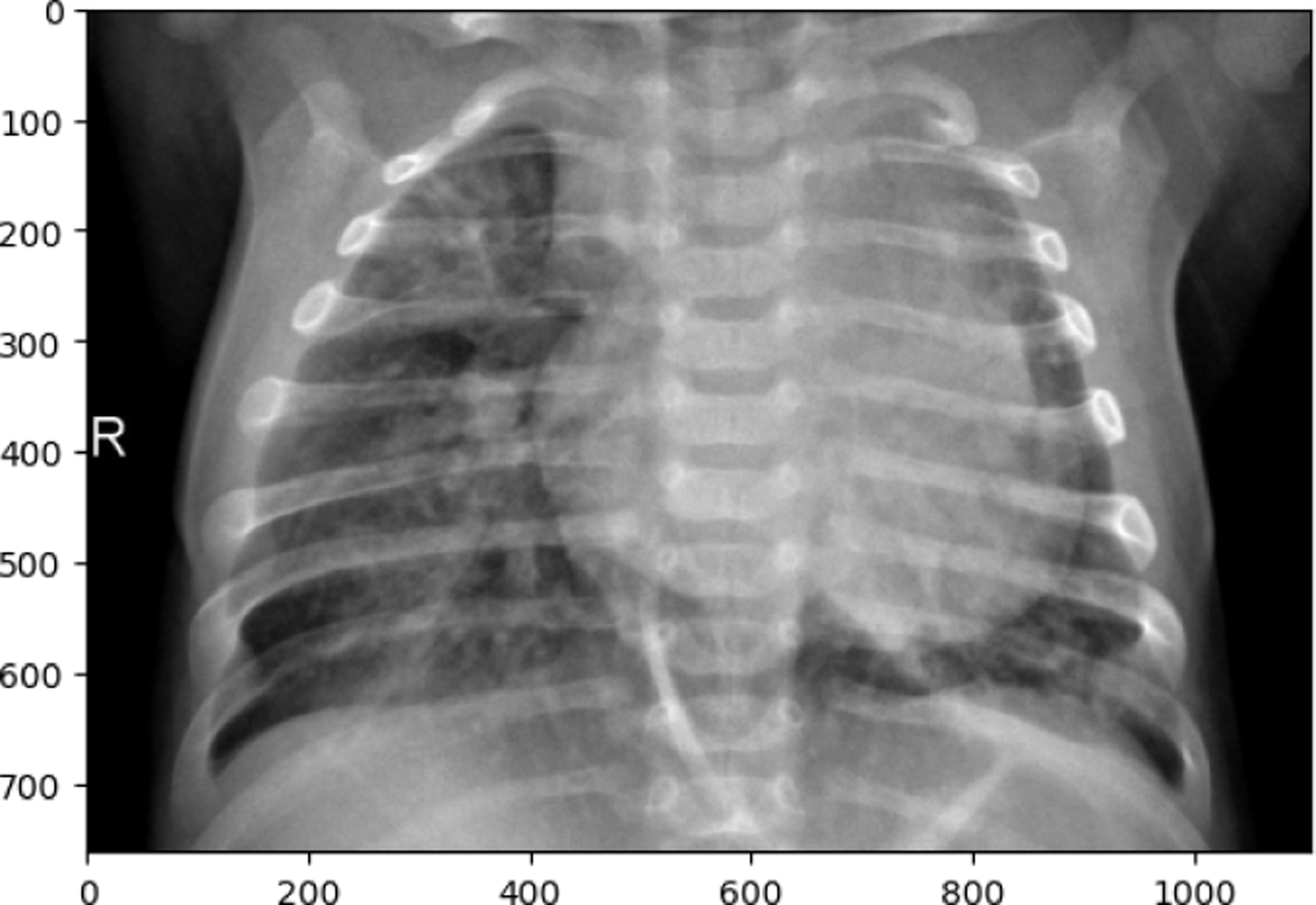
PNEUMONIA image in test set

**Figure 17.**
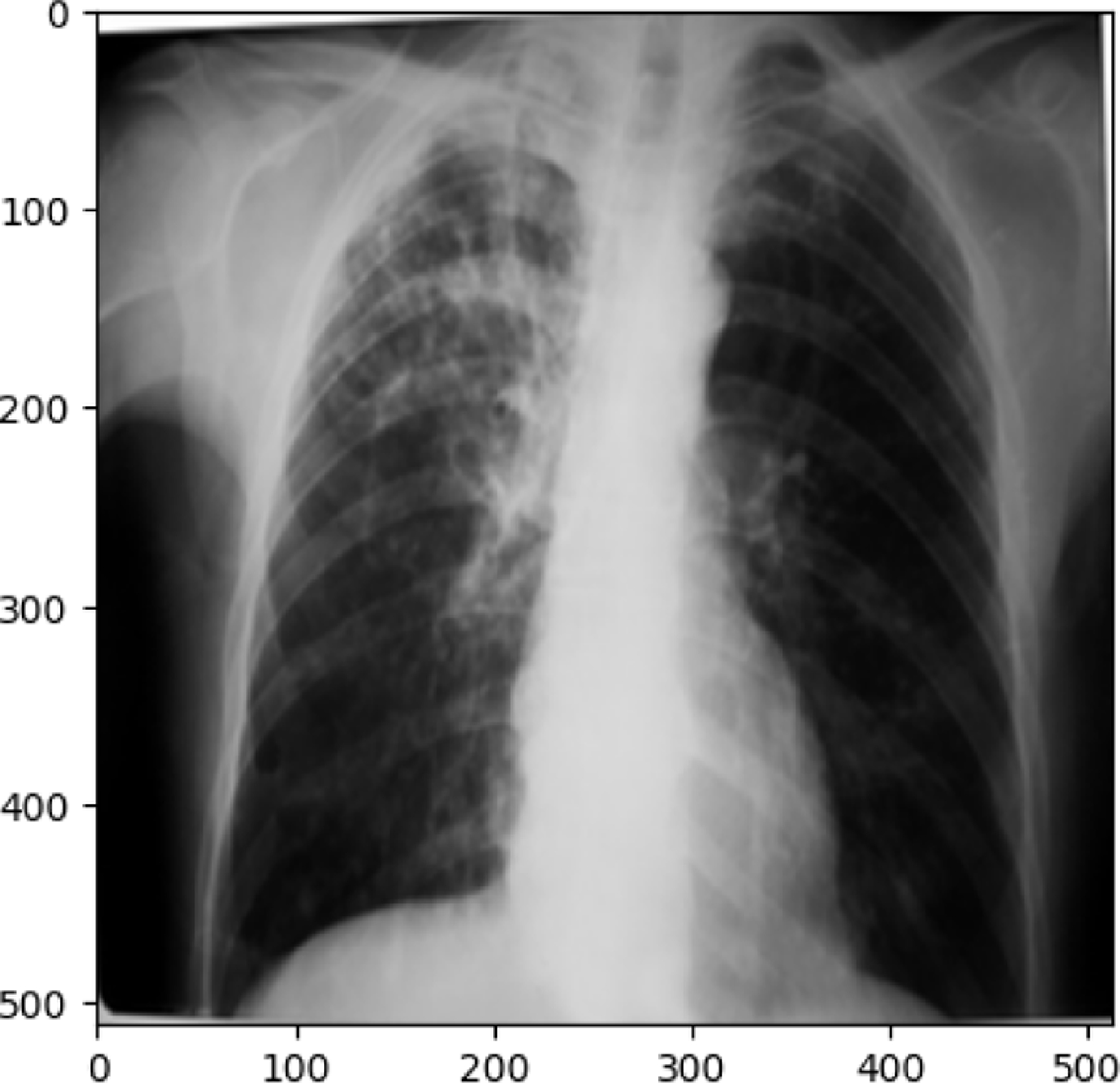
TUBERCULOSIS image in test set

**Figure 18.**
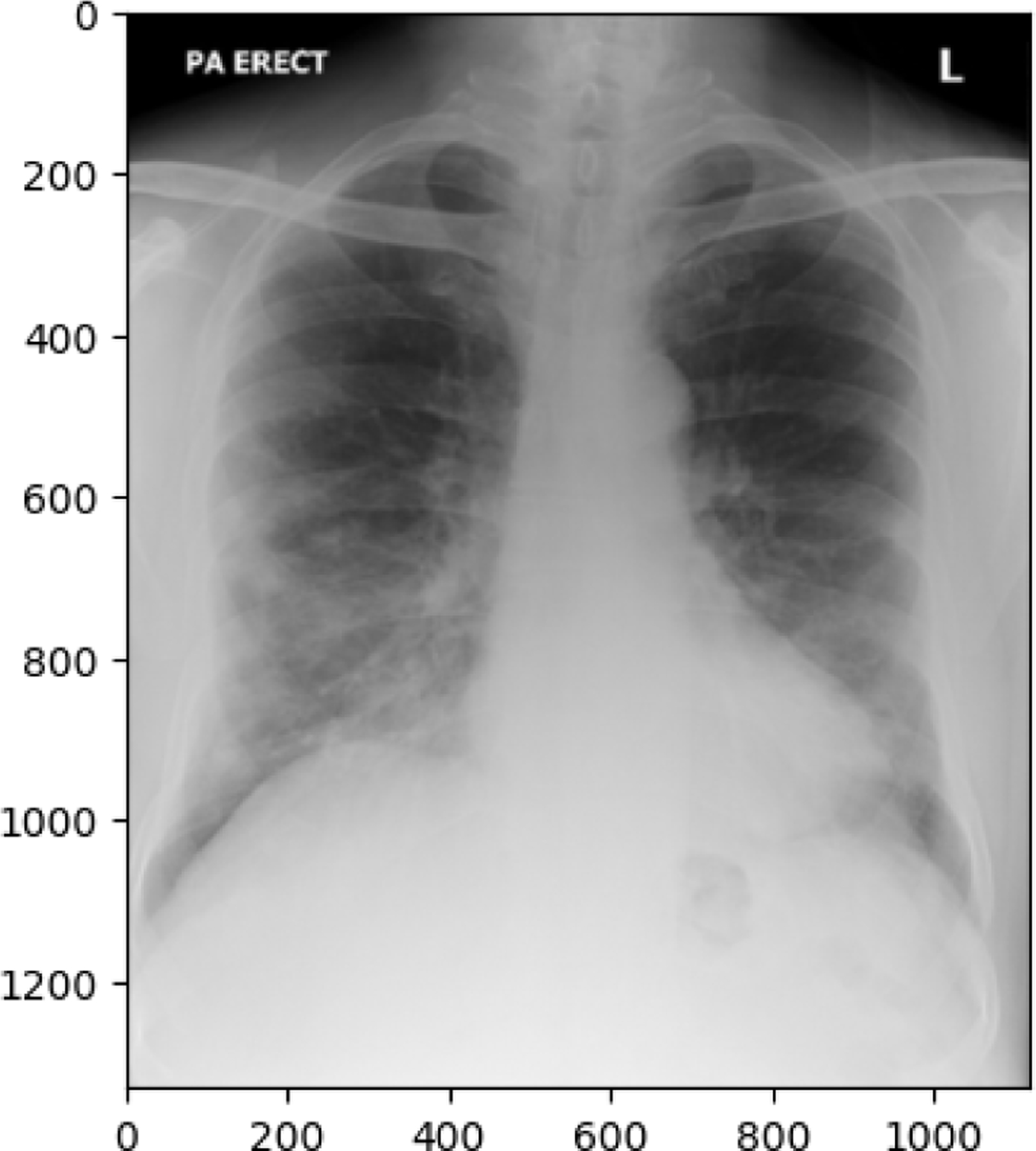
COVID19 image in val set

**Figure 19.**
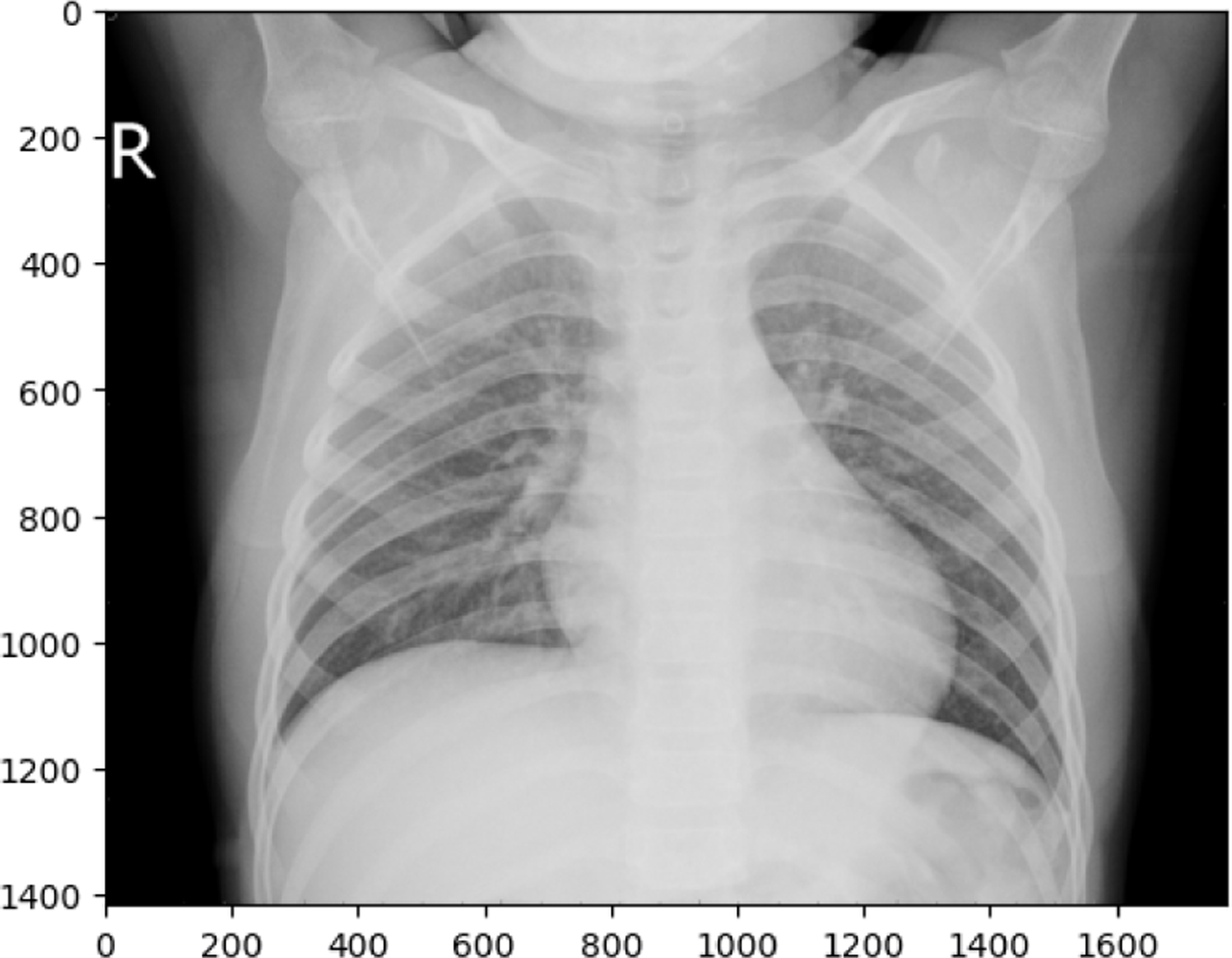
NORMAL image in val set

**Figure 20.**
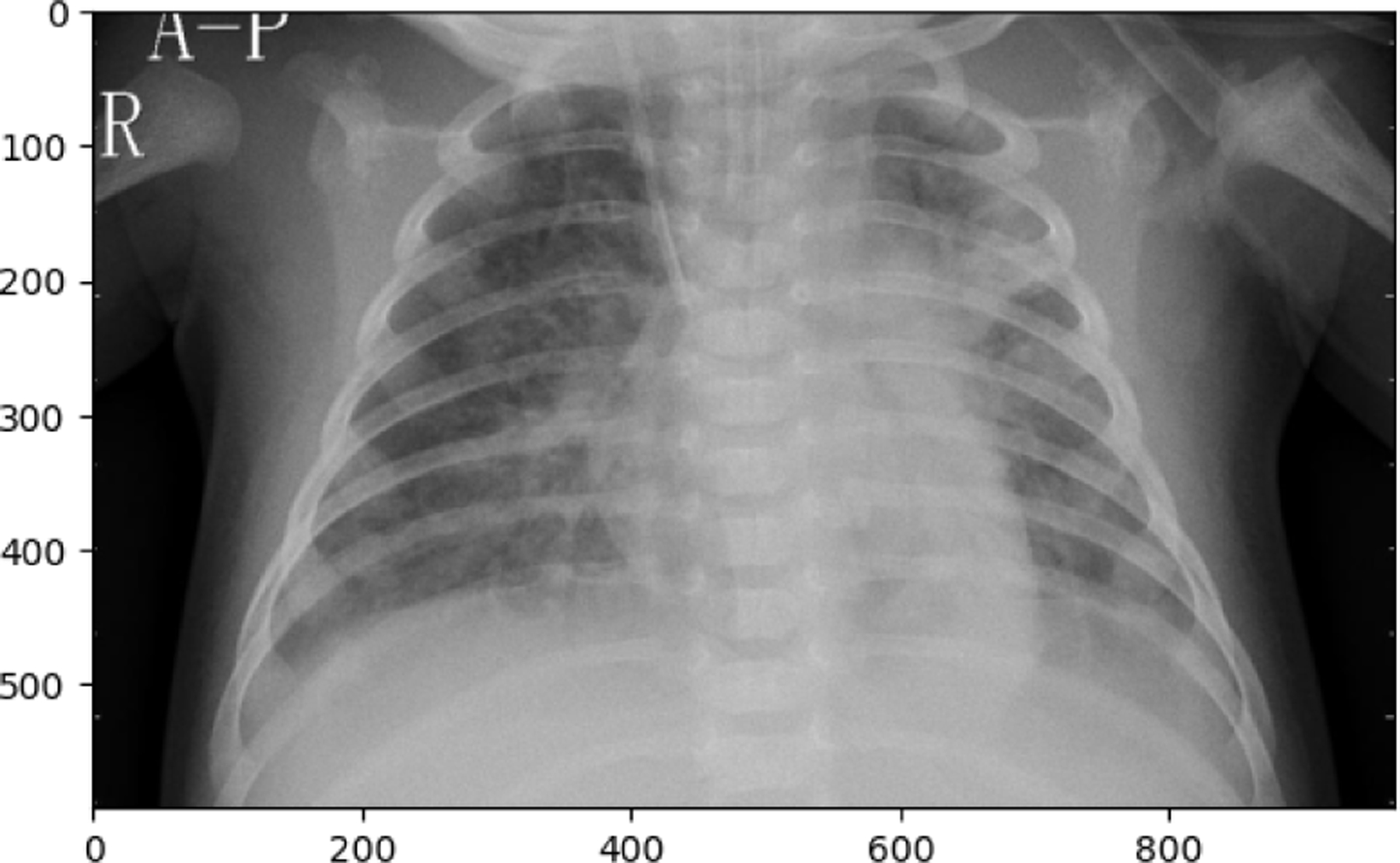
PNEUMONIA image in val set

**Figure 21.**
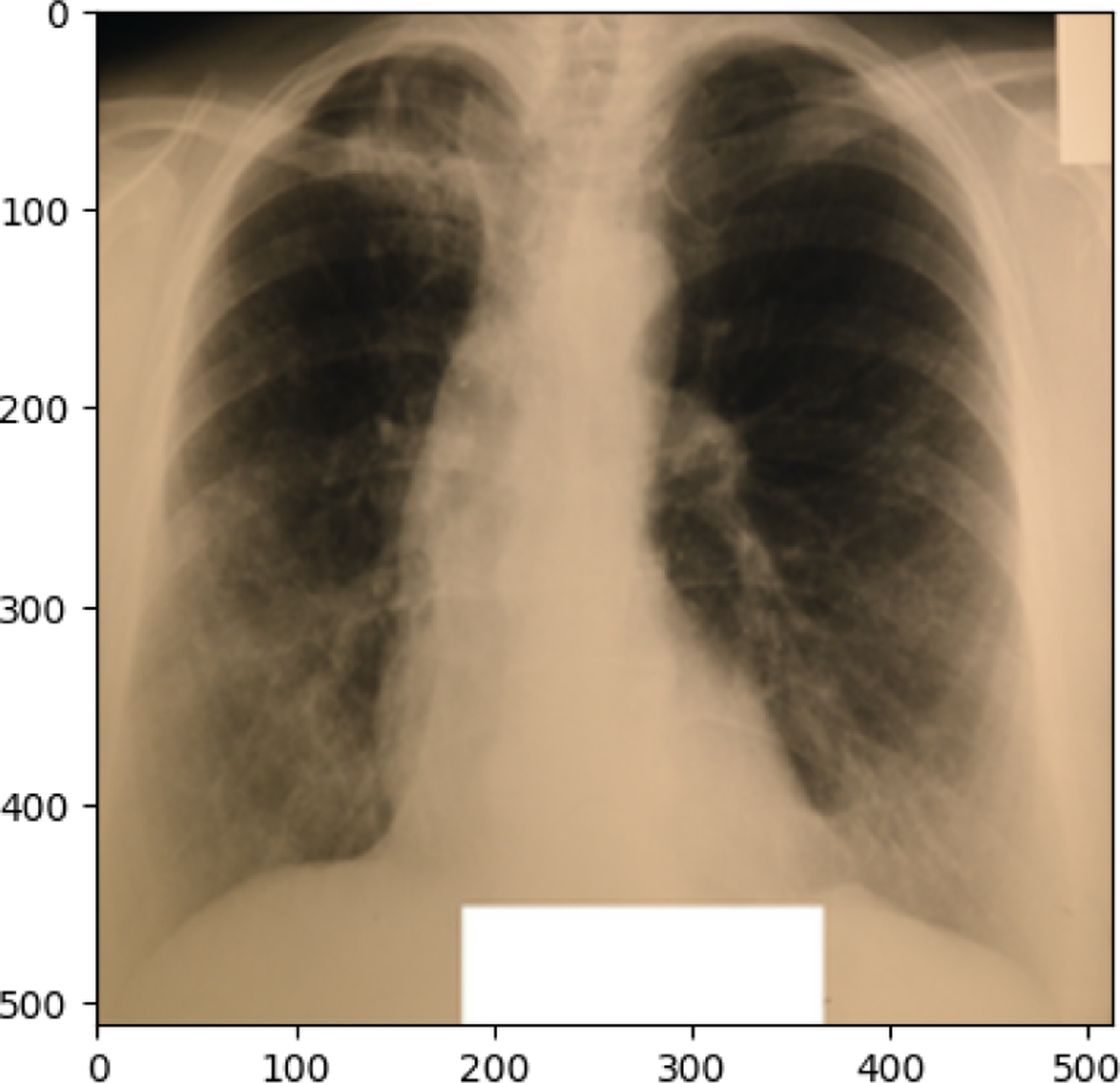
TUBERCULOSIS image in val set

**Figure 22.**
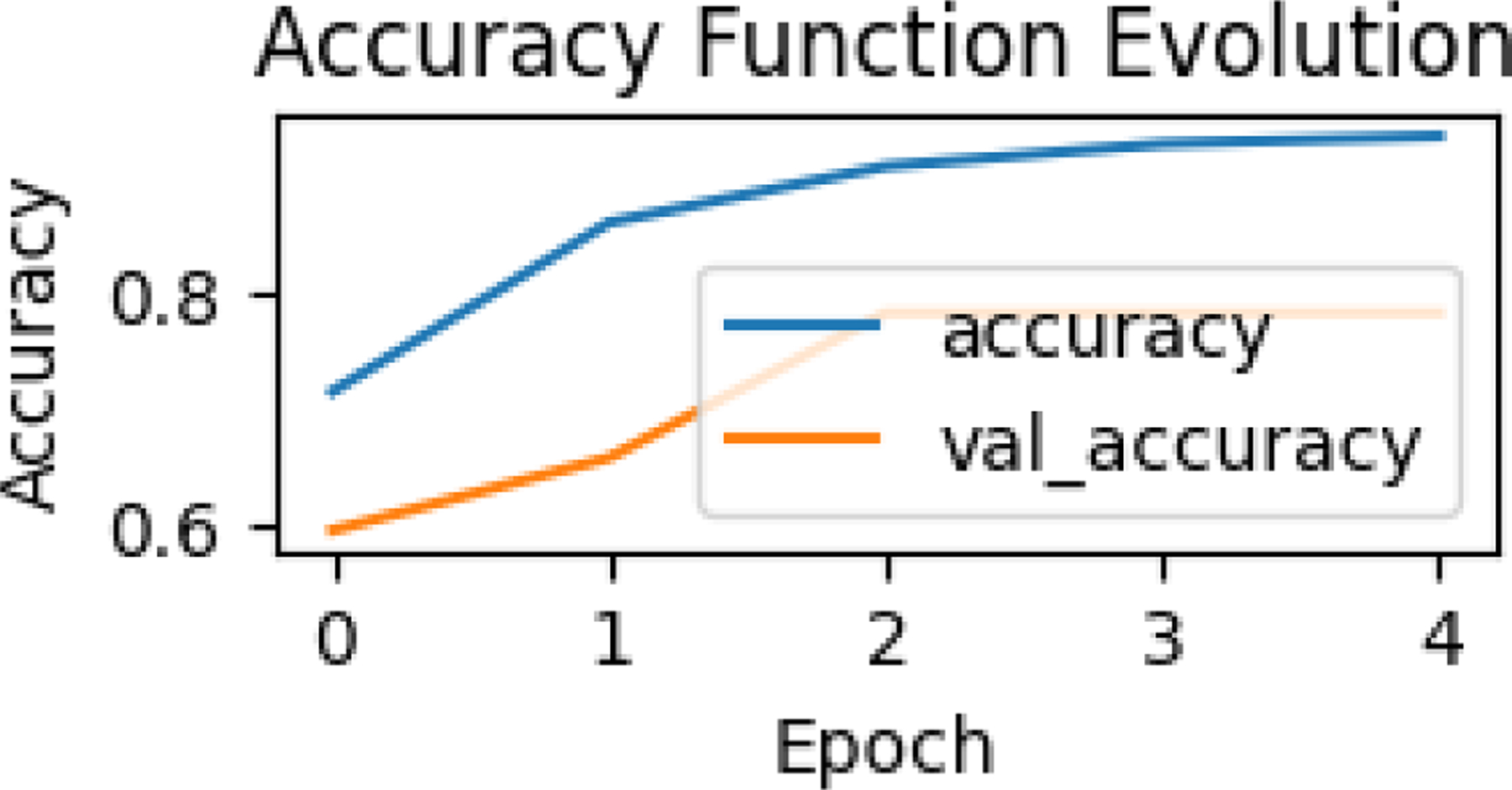
Accuracy Function Evolution

**Figure 23.**
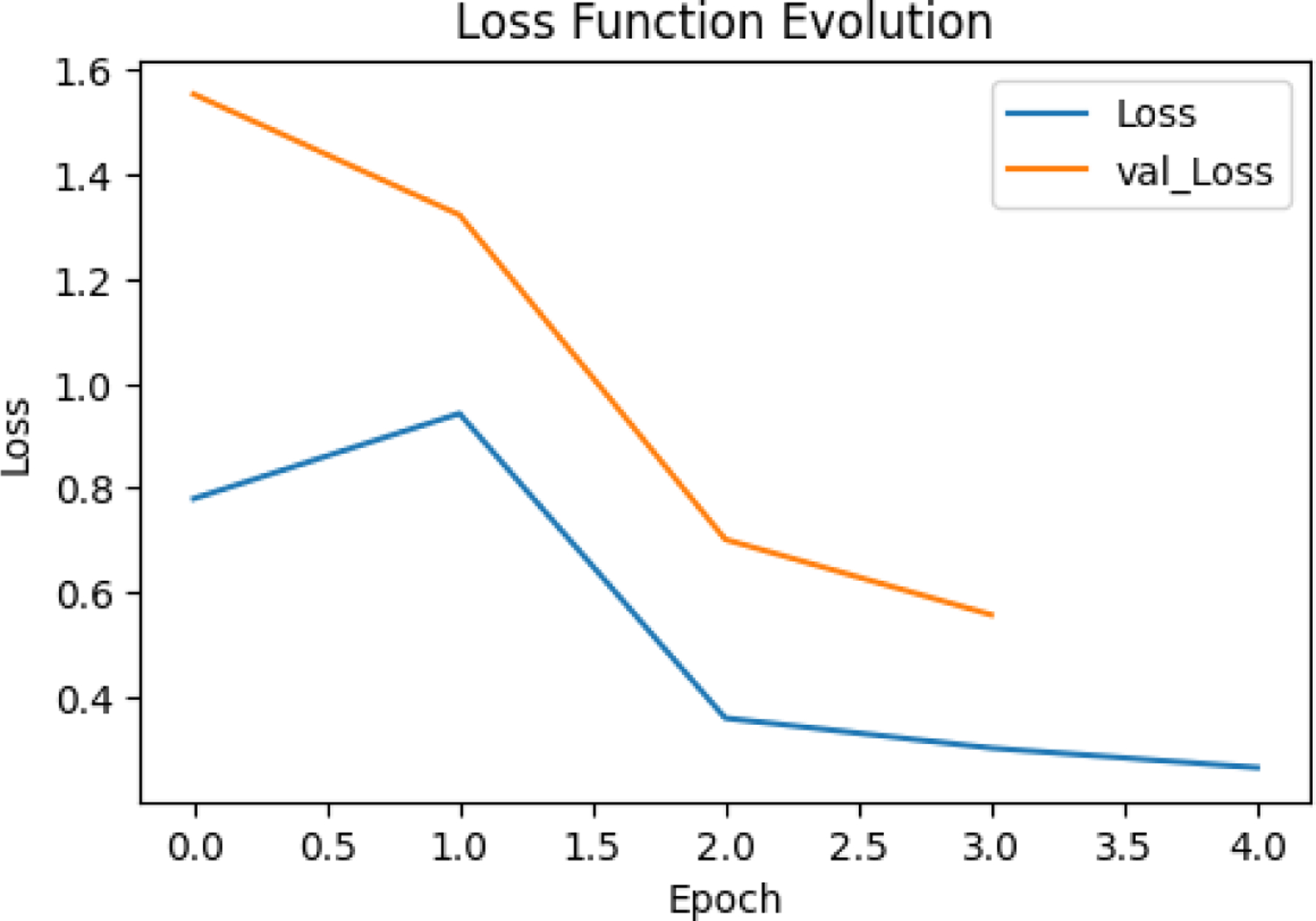
Loss function evolution

**Figure 24.**
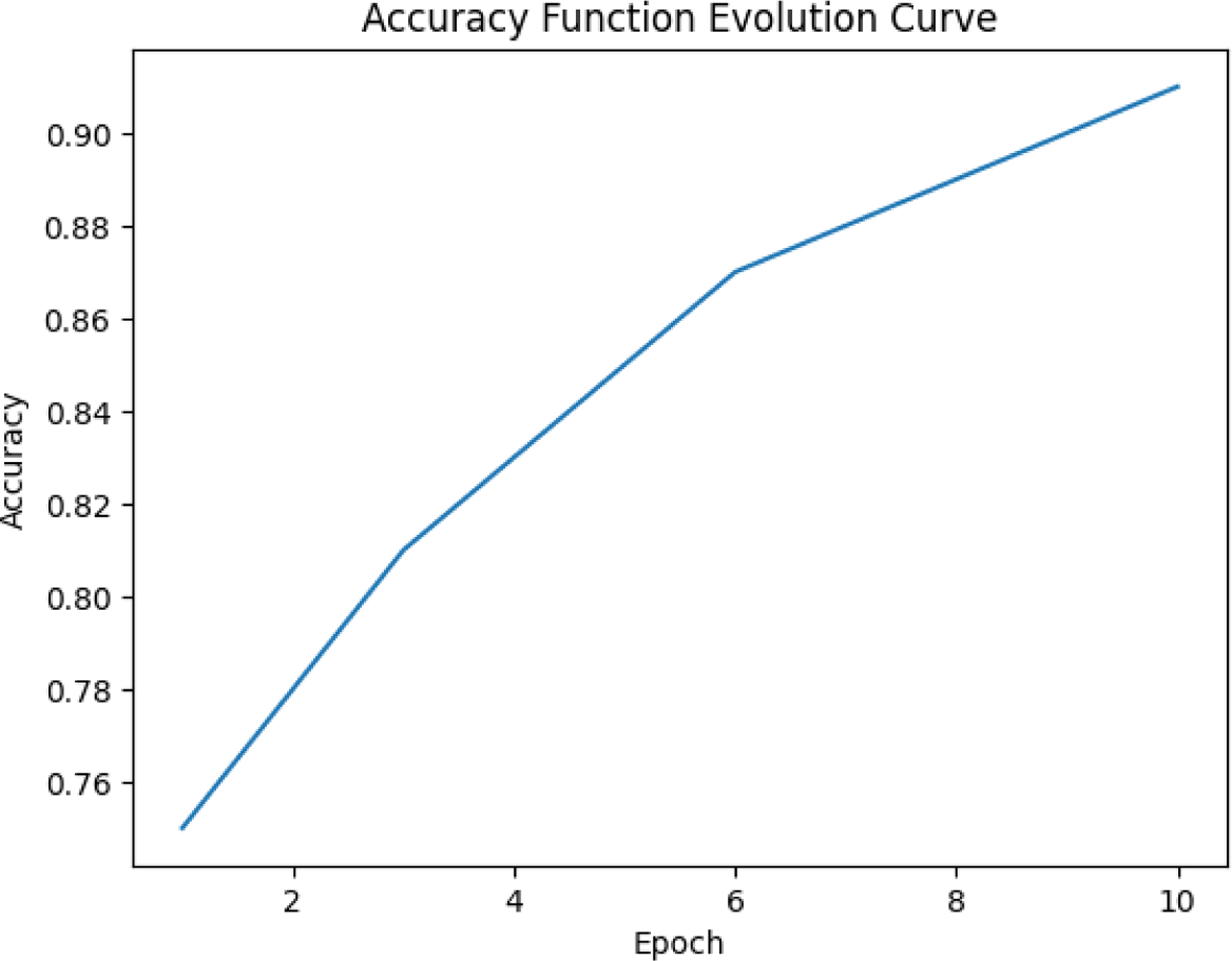
Loss function evolution

**Figure 25.**
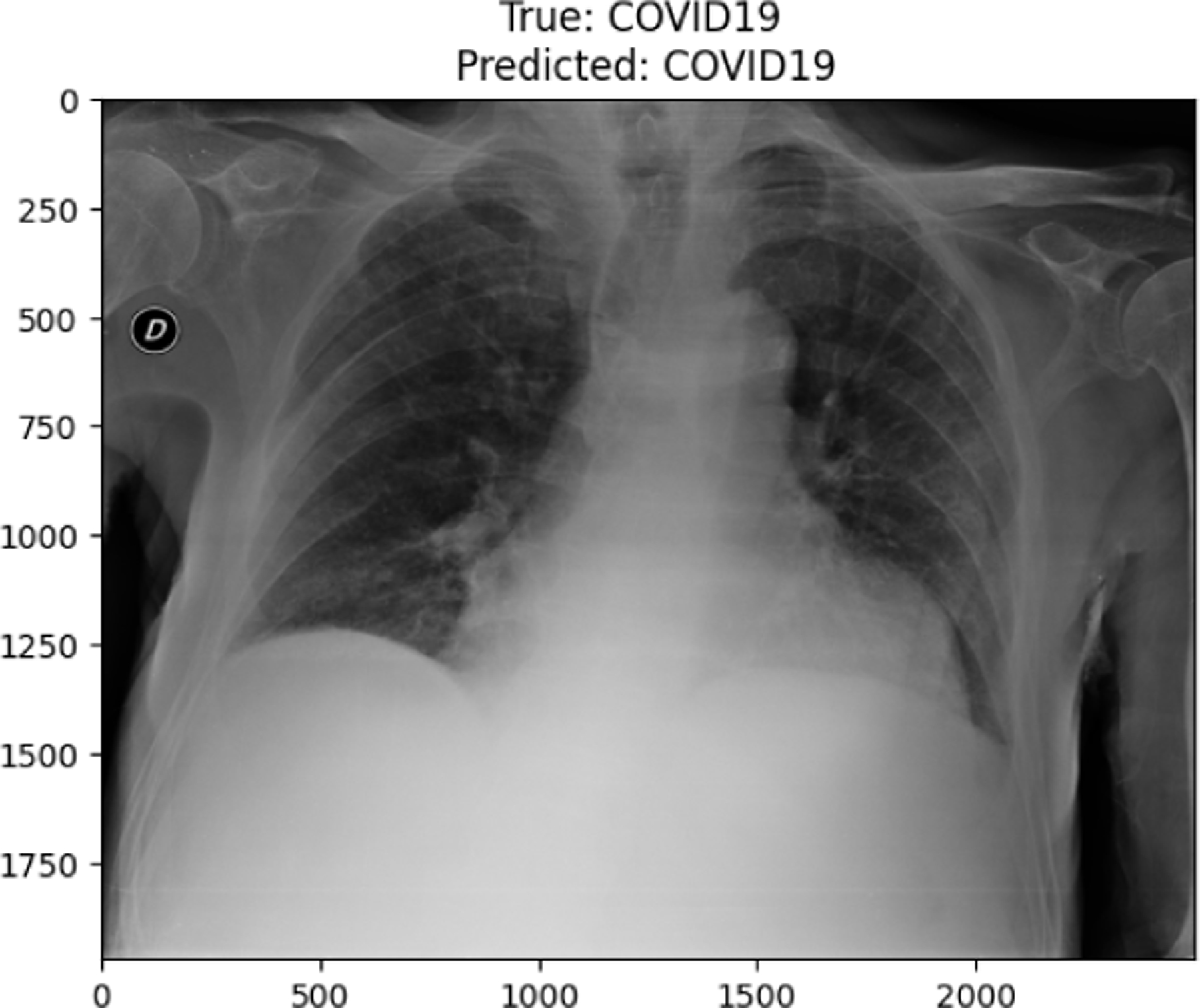
True Covid19 predicted as Covid19

**Figure 26.**
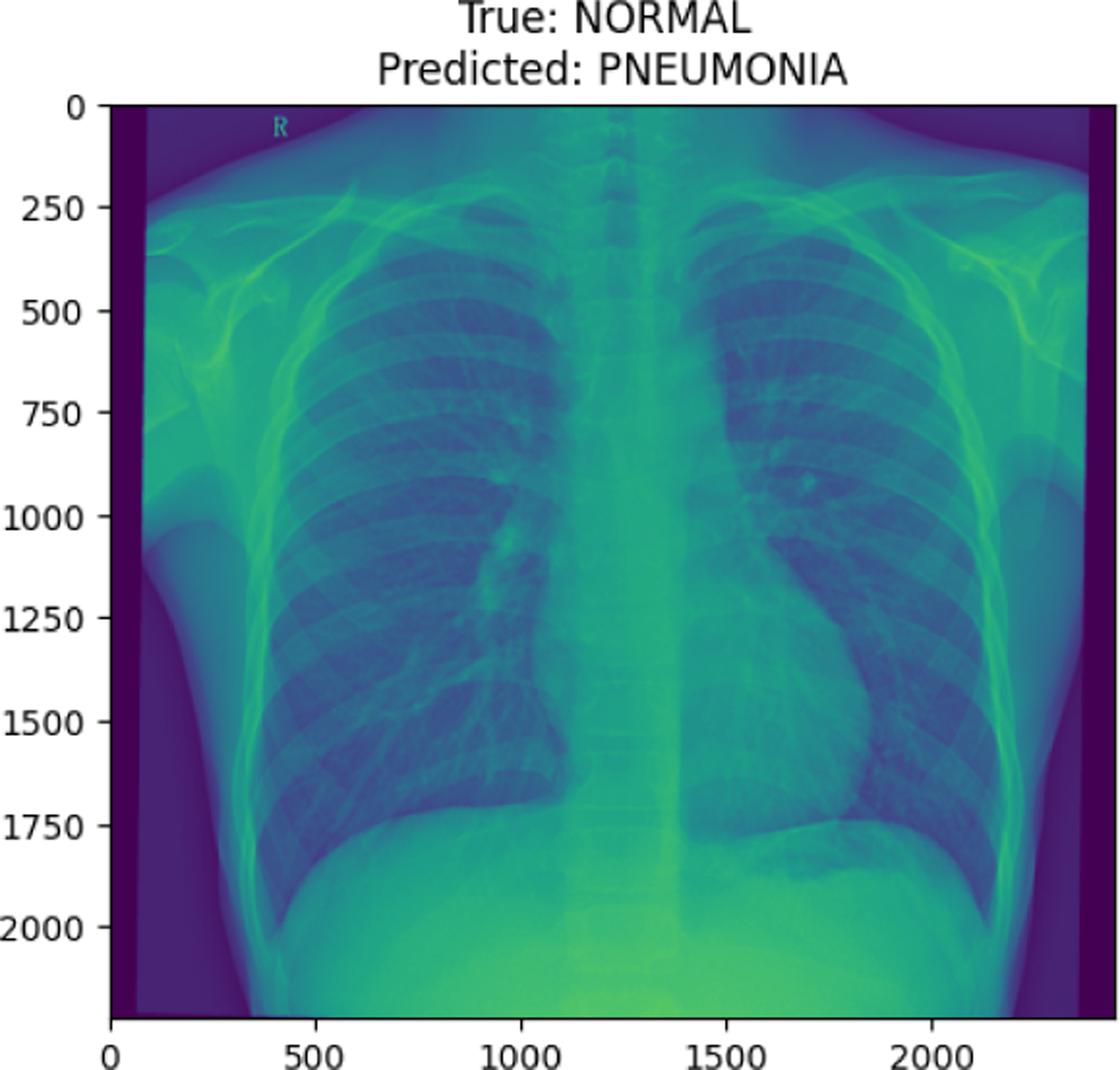
True Normal predicted as Pneumonia

**Figure 27.**
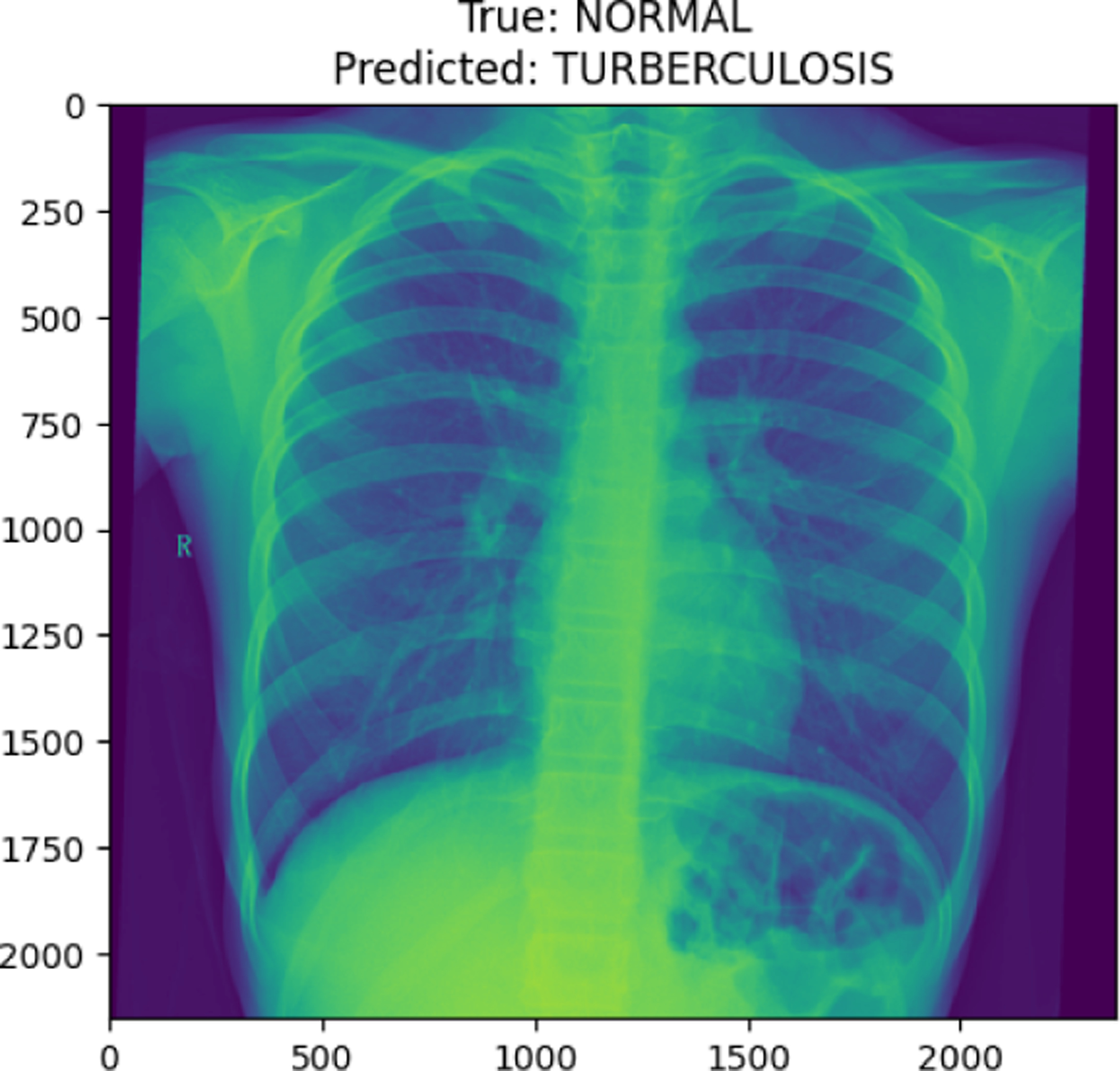
True Normal predicted as Tuberculosis

**Figure 28.**
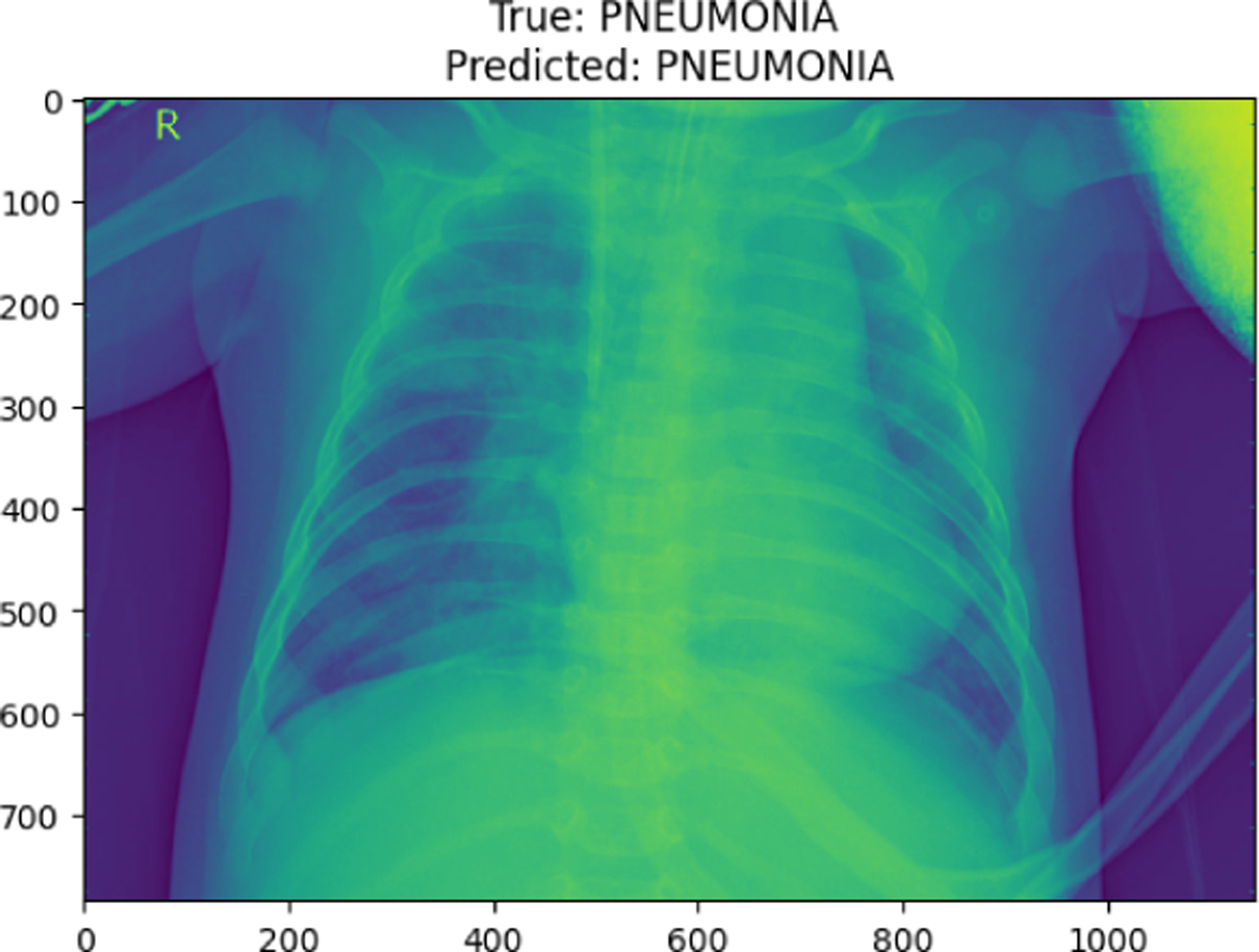
True Pneumonia predicted as Pneumonia

**Figure 29.**
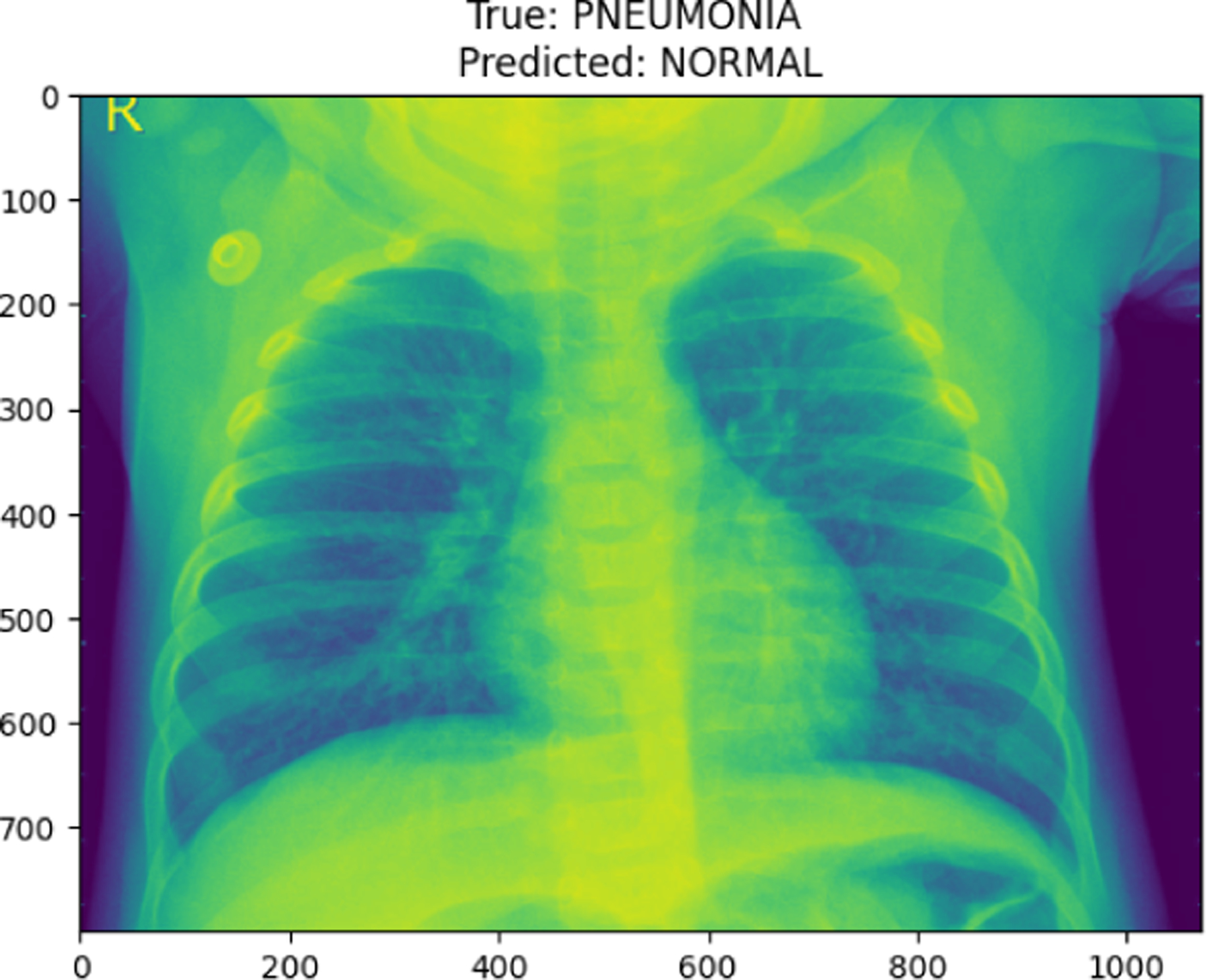
True Pneumonia predicted as Normal

**Figure 30.**
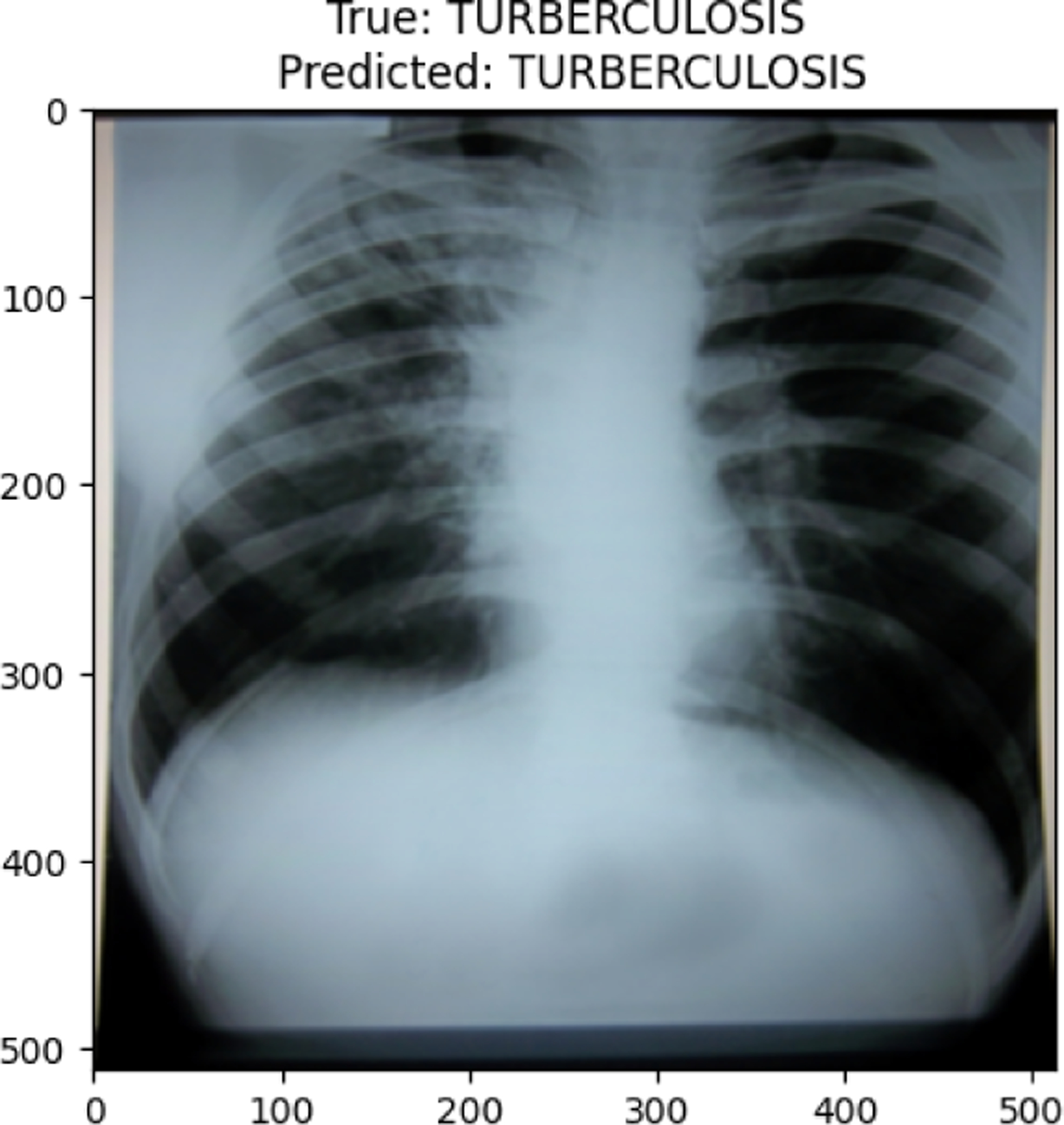
True Tuberculosis predicted as Tuberculosis

**Table 1.** Showing description of the used data set in the four categories

	Train set	Test set	Validation set	Total
COVID19	460	106	10	576
NORMAL	1341	234	8	1583
PNEUMONIA	3875	390	8	4273
TUBERCULOSIS	650	41	12	703
**TOTAL**	6326	771	38	7135

**Table 2.** Confusion Matrix for the Classification Model

Actual/Predicted	COVID19	NORMAL	PNEUMONIA	TUBERCULOSIS	Total
**COVID19**	98	0	8	0	106
**NORMAL**	0	80	150	4	234
**PNEUMONIA**	0	3	386	1	390
**TUBERCULOSIS**	0	0	3	38	41
**Total**	98	83	547	43	771

**Table 3.** Showing results of the performance metrics

CLASS	Precision	Recall	F1-score	Support
COVID19	0.80	0.92	0.85	106
NORMAL	0.98	0.34	0.51	234
PNEUMONIA	0.74	0.99	0.85	390
TUBERCULOSIS	0.84	0.93	0.88	41
accuracy			0.78	771
macro average	0.84	0.79	0.77	771
weighted average	0.83	0.78	0.75	771

**Table 4.** Neural Network Architecture Summary

Layer (Type)	Output Shape	Parameters
conv2d (Conv2D)	(None, 220, 220, 128)	3,328
max_pooling2d	(None, 73, 73, 128)	0
conv2d_1 (Conv2D)	(None, 69, 69, 64)	204,864
max_pooling2d_1	(None, 23, 23, 64)	0
conv2d_2 (Conv2D)	(None, 21, 21, 30)	17,310
max_pooling2d_2	(None, 10, 10, 30)	0
conv2d_3 (Conv2D)	(None, 8, 8, 30)	8,130
max_pooling2d_3	(None, 4, 4, 30)	0
flatten (Flatten)	(None, 480)	0
dense (Dense)	(None, 2048)	985,088
dense_1 (Dense)	(None, 512)	1,049,088
dropout (Dropout)	(None, 512)	0
dense_2 (Dense)	(None, 256)	131,328
dense_3 (Dense)	(None, 64)	16,448
dropout_1 (Dropout)	(None, 64)	0
dense_4 (Dense)	(None, 32)	2,080
dense_5 (Dense)	(None, 4)	132
**Total Parameters:**		**2,417,796**
**Trainable Parameters:**		**2,417,796**
**Non-trainable Parameters:**		**0**
